# Spiro-Linked Polyketides from Cultures of *Westerdykella dispersa* Ca4–13 as Inhibitors of iNOS-Associated Neuroinflammation

**DOI:** 10.1021/acs.jnatprod.6c00217

**Published:** 2026-05-28

**Authors:** Shu-Jung Huang, Su-Jung Hsu, Yi-Chien Liu, Cheng-Yan Jiang, George Hsiao, Tzong-Huei Lee

**Affiliations:** † Institute of Fisheries Science, 33561 National Taiwan University, Taipei 106319, Taiwan; ‡ Ph.D. Program in Drug Discovery and Development Industry, College of Pharmacy, 38032 Taipei Medical University, Taipei 110301, Taiwan; § Department of Pharmacology, School of Medicine, College of Medicine, Taipei Medical University, Taipei 110301, Taiwan; ∥ Graduate Institute of Medical Sciences, College of Medicine, Taipei Medical University, Taipei 110301, Taiwan; ⊥ Department of Life Science, College of Life Science, National Taiwan University, 106319 Taipei, Taiwan

## Abstract

Chemical investigation on fermented products by the fungal strain *Westerdykella dispersa* Ca4–13 isolated from edible oysters *Crassostrea angulata* collected from Taiwan resulted in the isolation of seven chemical entities. Their structures were elucidated by spectroscopic analysis to be westeroic acid A (**1**), westeroic acid B (**2**), westeroic acid C (**3**), auranticin A (**4**), auranticin B (**5**), pilobolusone C (**6**), and epi-radicinol (**7**). Among these, westeroic acid A (**1**) is a novel C_14_ polyketide with a γ-lactone functionality, while westeroic acids B (**2**) and C (**3**) are two rare C_28_ polyketides with a unique 6/6-spiro-linked δ-lactone moiety. Compounds **1**, **2**, **3**, and **7** exhibited anti-inflammatory activities on nitric oxide production in lipopolysaccharide (LPS)-induced BV-2 microglial cells with IC_50_ values ranging from 9.9 to 11.3 μM. Compounds **2** and **3** significantly suppressed LPS-induced inducible nitric oxide synthase (iNOS) protein expression. Molecular docking analysis using murine iNOS (PDB ID: 1QW4) provided structural insight into these observations, suggesting that effective inhibition was associated with cooperative interaction networks within the l-arginine binding pocket rather than a single dominant interaction. Overall, these findings highlight their promise as potential lead compounds for further neuroinflammation-related drug development.

## Graphical abstract

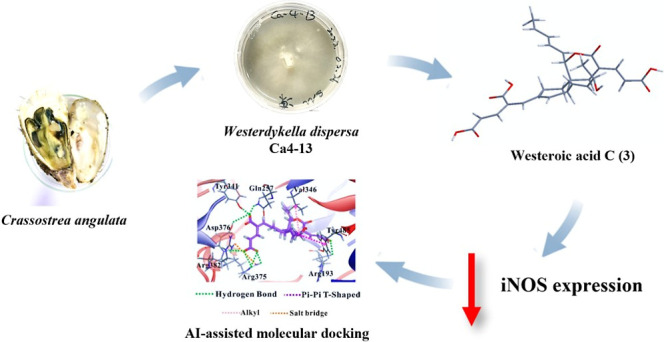

Marine-derived fungi represent a prolific source of structurally diverse secondary metabolites, many of which exhibit potent biological activities and continue to inspire drug discovery efforts. In recent years, marine invertebrates and their associated microbiomes have gained attention as promising yet underexplored reservoirs of chemically novel fungal taxa.^1–3^ Among the hosts, oysters (*Crassostrea* spp.) provide a unique ecological niche shaped by their filter-feeding lifestyle, which supports complex microbial communities.^4–6^ However, despite growing interest, the metabolic potential and chemical diversity of oyster-associated fungi remain largely underexplored. Previous investigations have emphasized the potential of marine fungal metabolites as modulators of inflammatory responses and neuroprotective agents.^7–9^ In our recent study, the fungal strain *Westerdykella dispersa* Ca4–13, isolated from the edible oyster *Crassostrea angulata* collected in Taiwan, was found to produce cytochalasans exhibiting significant inhibitory effects on nitric oxide (NO) production in BV-2 microglial cells.^10^ Given that microglia-mediated neuroinflammation is as a key pathogenic mechanism in neurodegenerative disorders, the identification of additional fungal metabolites capable of regulating NO production and inducible nitric oxide synthase (iNOS) expression remains of considerable interest.^11–13^ To further probe the chemical diversity of *W. dispersa* Ca4–13, the OSMAC (one strain, many compounds) strategy was applied to promote secondary metabolic diversification under varied culture conditions. Bioassay-guided fractionation led to the isolation and characterization of seven metabolites **1**–**7**, including three previously unreported polyketides. Their antineuroinflammatory activities were evaluated using lipopolysaccharide-induced BV-2 microglial cells, and molecular docking analyses were subsequently conducted to provide mechanistic insight into ligand–iNOS interactions.

## Results and Discussion

The EtOAc extract of the fermented broth of *W. dispersa* Ca4–13 was fractionated and purified sequentially by column chromatography on Sephadex LH-20 and semipreparative HPLC to afford compounds **1**–**3** along with auranticin A (**4**),^14^ auranticin B (**5**),^14^ pilobolusone C (**6**),^15^ and epi-radicinol (**7**).^16^ 
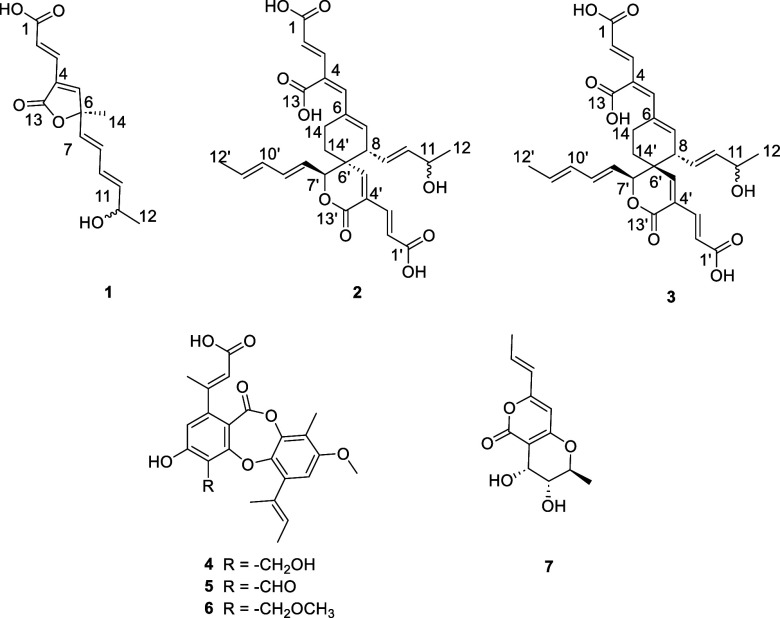


Compound **1** was obtained as pale yellow powder, and determined to have the molecular formula C_14_H_16_O_5_ as deduced from a deprotonated molecular ion [M – H]^−^ at *m*/*z* 263.0914 (calcd. 263.0924 for C_14_H_15_O_5_) in the HRESIMS (Figure S1), supported by analysis of ^13^C NMR data (Table 1andFigure S4), indicating 7 degrees of unsaturation. Its IR spectrum provided evidence for the presence of a conjugated carboxylic acid (3380–2927 and 1699 cm^–1^) and a conjugated γ-lactone moiety (1748 cm^–1^) (Figure S2). Analysis of ^1^H NMR and HSQC spectra of **1** (Figures S3 and S5) indicated signals for two methyl groups at δ_H_ 1.23 (3H, d, *J* = 6.6 Hz, H_3_-12) and 1.59 (3H, s, H_3_-14); seven olefinic protons at δ_H_ 5.74 (1H, d, *J* = 15.2 Hz, H-7), 5.84 (1H, dd, *J* = 15.2, 6.6, Hz, H-10), 6.21 (1H, ddd, *J* = 15.2, 10.5, 1.3 Hz, H-9), 6.33 (1H, dd, *J* = 15.2, 10.5 Hz, H-8), 6.92 (1H, d, *J* = 16.5 Hz, H-2), 7.33 (1H, d, *J* = 16.5 Hz, H-3), and 7.82 (1H, s, H-5); and one oxygenated methine at δ_H_ 4.28 (1H, qdd, *J* = 6.6, 6.6, 1.3 Hz, H-11) (Table 2).^17^ Interpretation of ^13^C NMR and HSQC spectra revealed 14 carbon signals attributable to two methyl groups at δ_C_ 23.5 (C-12) and 24.3 (C-14); one oxygenated methine at δ_C_ 68.7 (C-11); seven olefinic methines at δ_C_ 126.2 (C-2), 128.8 (C-9), 130.9 (C-7), 132.5 (C-8), 133.6 (C-3), 141.6 (C-10), and 160.3 (C-5); and four nonprotonated carbons at δ_C_ 87.4 (C-6), 127.1 (C-4), 169.9 (C-1), and 172.0 (C-13). COSY correlations of H-2/H-3, H-7/H-8, H-8/H-9, H-9/H-10, H-10/H-11, and H-11/H_3_-12 (Figures 1andS6), together with key HMBC cross-peaks (Figure S7) of H-2/C-1, H-3/C-1 and C-4, H-5/C-4 and C-6, H-7/C-6, and H-8/C-6, established the sequential connectivity of the C-1–C-12 carbon chain.^18^ HMBC correlations from H_3_-14 to C-5, C-6, and C-7 further confirmed the attachment of C-14 to this framework. Seven degrees of unsaturation of **1**, including four double bonds at Δ^2^, Δ^4^, Δ^7^, and Δ^9^ together with a carboxylic acid at C-1 and a carbonyl group at C-13, in combination with key HMBC correlations from H-5 to C-4, C-6, and C-13, and the mesomeric downfield shift of H-5/C-5 at respective δ_H‑5_ 7.82/δ_C‑5_ 160.3, supported the presence of an additional γ-lactone ring formed by O–C-6–C-5–C-4–C-13–O. Based on above spectroscopic analysis, the planar structure of compound **1** was established. The configurations of the Δ^2^, Δ^7^, and Δ^9^ double bonds were assigned as *E* based on the coupling constants of *J*_H‑2/H‑3_ (16.5 Hz), *J*_H‑7/H‑8_ (15.2 Hz), and *J*_H‑9/H‑10_ (15.2 Hz), respectively. NOESY spectrum of compound **1** (Figure S8) could not assist in determining the stereochemistry at C-6 and C-11. The experimental electronic circular dichroism (ECD) spectrum of compound **1** exhibited a positive Cotton effect at 205 nm (θ + 99.6 mdeg) and a negative Cotton effect at 241 nm (θ–29.3 mdeg). The TDDFT-calculated –ECD spectrum for the 6*R*,11*R*-stereoisomer with a positive Cotton effect at 205 nm and a negative Cotton effect at 244 nm showed good agreements in the sign and overall shape of the Cotton effects of the experimental spectrum, suggesting the 6*R*,11*R* absolute configurations of **1** (Figure 2A). However, the overall trend of the 6*R*,11*S* isomer is also quite similar, making it difficult to clearly distinguish between these two possibilities. Therefore, only the absolute configuration at C-6 could be confidently assigned as *R* form. Compound **1** was named westeroic acid A.

**Table 1:** ^13^C NMR Spectroscopic Data for Compounds **1**–**3** ^a^

	1^a^	2^a^	3^a^
pos	δ_C_, type	δ_C_, type	δ_C_, type
1	169.9, C	170.1, C	170.0, C
2	126.2, CH	120.9, CH	120.9, CH
3	133.6, CH	145.3, CH	145.2, CH
4	127.1, C	134.0, C	133.5, C
5	160.3, CH	141.0, CH	141.2, CH
6	87.4, C	136.9, C	136.3, C
7	130.9, CH	136.8, CH	136.1, CH
8	132.5, CH	45.9, CH	49.3, CH
9	128.8, CH	128.0, CH	128.4, CH
10	141.6, CH	141.1, CH	140.3, CH
11	68.7, CH	69.0, CH	69.2, CH
12	23.5, CH_3_	23.8, CH_3_	23.5, CH_3_
13	172.0, C	172.2, C	171.9, C
14	24.3, CH_3_	23.5, CH_2_	22.6, CH_2_
1′		170.1, C	170.2, C
2′		123.7, CH	123.6, CH
3′		139.9, CH	139.8, CH
4′		128.8, C	128.8, C
5′		155.5, CH	154.3, CH
6′		43.0, C	42.2, C
7′		84.7, CH	84.1, CH
8′		122.8, CH	124.5, CH
9′		139.1, CH	138.9, CH
10′		131.6, CH	131.5, CH
11′		134.0, CH	134.1, CH
12′		18.4, CH_3_	18.4, CH_3_
13′		164.3, C	164.1, C
14′		27.7, CH_2_	27.6, CH_2_

a Measured in MeOH-*d*_4_ (150 MHz).

**Table 2:** ^1^H NMR Spectroscopic Data for Compounds **1**–**3**

	1^a^	2^a^	3^a^
pos	δ_H_, mult (*J* in Hz)	δ_H_, mult (*J* in Hz)	δ_H_, mult (*J* in Hz)
2	6.92, d (16.5)	5.89, d (15.0)	5.88, d (15.0)
3	7.33, d (16.5)	7.29, d (15.0)	7.29, d (15.0)
5	7.82, s	6.50, s	6.52, s
7	5.74, d (15.2)	5.90^b^	5.98, d (2.8)
8	6.33, dd (15.2, 10.5)	3.13, m	3.04, m
9	6.21, ddd (15.2, 10.5, 1.3)	5.52, dd (15.3, 8.6)	5.56, dd (15.3, 8.6)
10	5.84, dd (15.2, 6.6)	5.63, dd (15.3, 6.5)	5.51, dd (15.3, 6.4)
11	4.28, qdd (6.6, 6.6, 1.3)	4.24, qd (6.5, 6.5)	4.16, qd (6.4, 6.4)
12	1.23, d (6.6)	1.22, d (6.5)	1.15, d (6.4)
14	1.59, s	2.26, m	2.40, br s
		2.37, m	
2′		6.66, d (16.0)	6.67, d (16.0)
3′		7.31, d (16.0)	7.32, d (16.0)
5′		7.22, s	7.00, s
7′		4.96, d (8.5)	4.90^c^
8′		5.66, dd (15.0, 8.5)	5.66, dd (15.0, 9.1)
9′		6.43, dd (15.0, 10.5)	6.34, dd (15.0, 10.5)
10′		6.17, dd (15.0, 10.5)	6.08, dd (15.0, 10.5)
11′		5.88^b^	5.85, m
12′		1.79, d (6.7)	1.75, d (6.7)
14′		1.76, m	1.76, m
		1.98, m	1.82, m

a Measured in MeOH-*d*_4_ (600 MHz).

b These signals were mutually overlapped, and assigned from the HSQC spectrum.

c This signal was overlapped with H_2_O resonance, and it was assigned from the HSQC spectrum.

**Figure 1:**
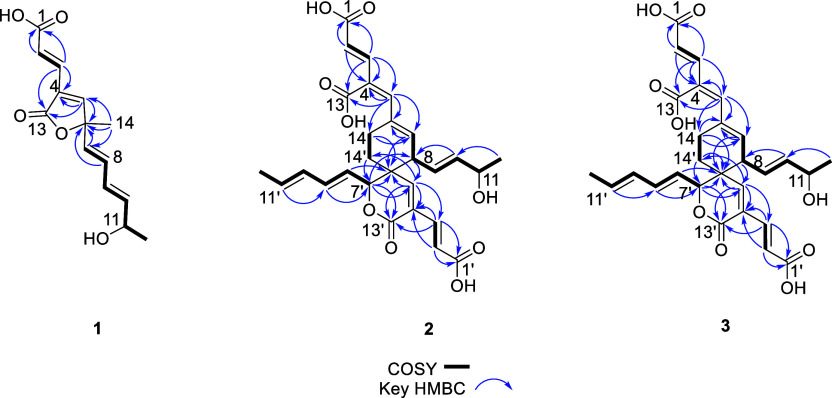
Key HMBC and COSY correlations of compounds **1**–**3**.

**Figure 2:**
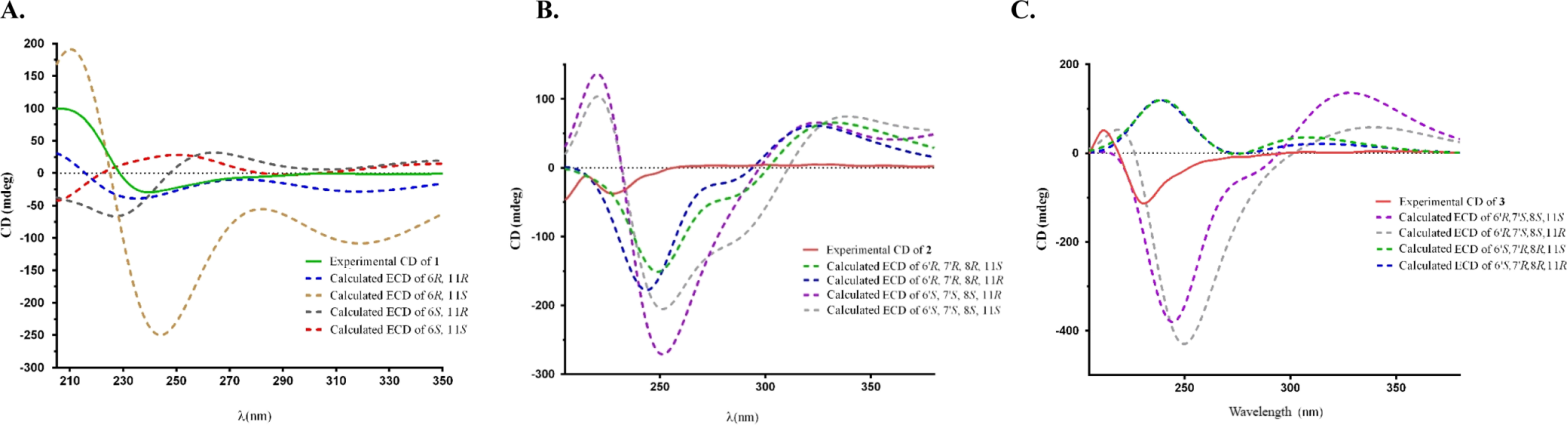
Experimental and calculated ECD spectra of compounds **1**–**3** (**A**–C, respectively).

**Figure 3:**
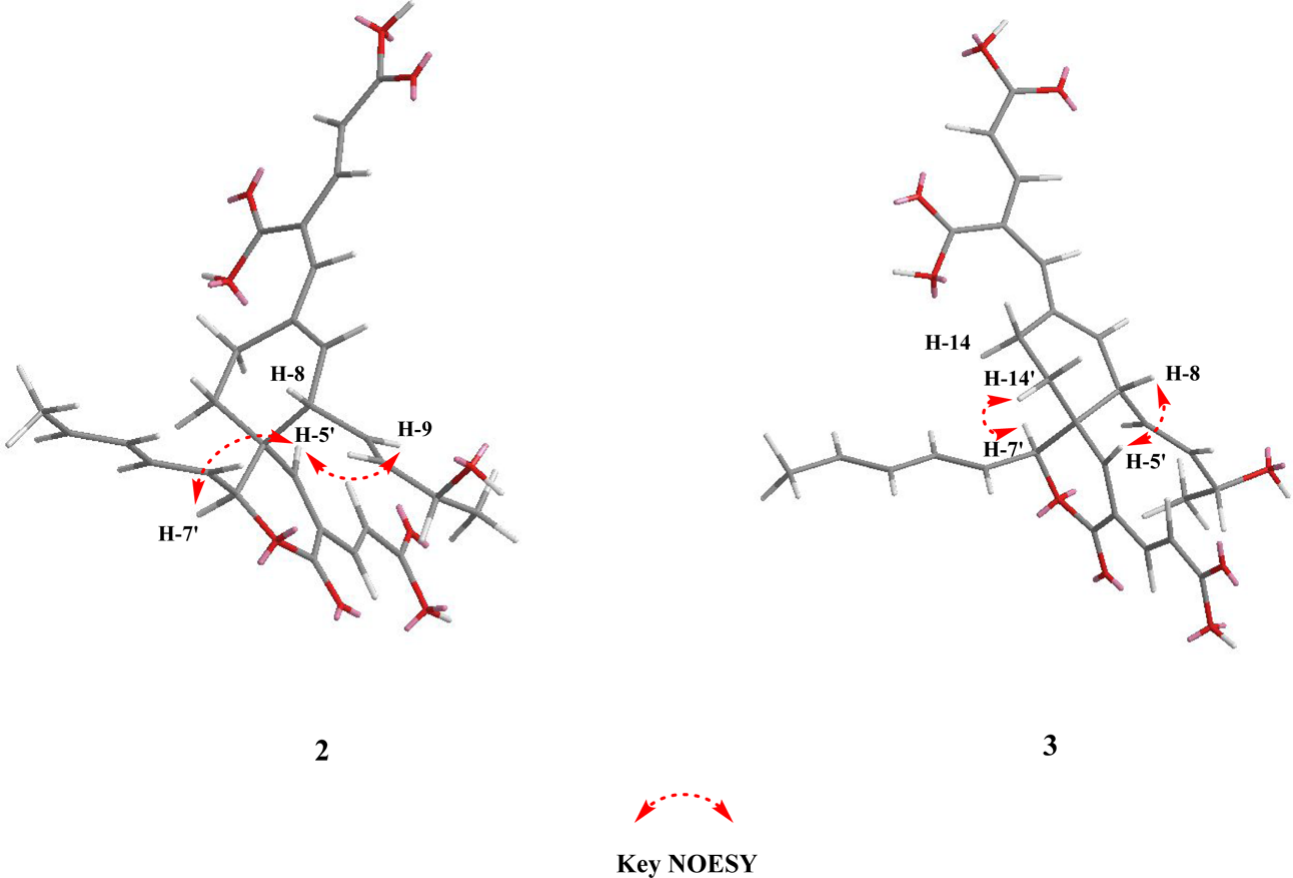
Energy-minimized molecular models of compounds **2** and **3** generated by ChemBio3D Ultra 14.0, illustrating the relative spatial orientations of the substituents, which are consistent with the observed NOESY correlations.

Compound **2** was isolated as yellow powder, and presented the molecular formula C_28_H_30_O_9_ deduced from a deprotonated molecular ion [M – H]^−^ at *m*/*z* 509.1817 (calcd 509.1817 for C_28_H_29_O_9_) in the HRESIMS (Figure S9), indicating 14 degrees of unsaturation. Its IR spectrum revealed the presence of a conjugated carboxylic acid (3344–2337 and 1662 cm^–1^) and a conjugated δ-lactone moiety (1709 cm^–1^) (Figure S10). Analysis of ^1^H NMR and HSQC spectra of **2** (Figures S11 and S13) indicated signals for two methyl groups at δ_H_ 1.22 (3H, d, *J* = 6.5 Hz, H_3_-12), 1.79 (3H, d, *J* = 6.7 Hz, H_3_-12′); two methylenes at δ_H_ 1.76 (1H, m, H_a_-14′) and 1.98 (1H, m, H_b_-14′) and 2.26 (1H, m, H_a_-14) and 2.37 (1H, m, H_b_-14); one aliphatic methine at δ_H_ 3.13 (1H, dd, *J* = 8.6, 2.8 Hz, H-8); two oxygenated methines at δ_H_ 4.24 (1H, qd, *J* = 6.5, 6.5 Hz, H-11) and 4.96 (1H, d, *J* = 8.5 Hz, H-7′); 13 olefinic protons at δ_H_ 5.52 (1H, dd, *J* = 8.6, 6.5 Hz, H-9), 5.63 (1H, dd, *J* = 8.6, 6.5 Hz, H-10), 5.66 (1H, dd, *J* = 15.0, 8.5 Hz, H-8′), 5.88 (1H, overlapped, H-11′), 5.89 (1H, d, *J* = 15.0 Hz, H-2), 5.90 (1H, overlapped, H-7), 6.17 (1H, dd, *J* = 15.0 Hz, 10.5, H-10′), 6.43 (1H, dd, *J* = 15.0, 10.5 Hz, H-9′), 6.50 (1H, s, H-5), 6.66 (1H, d, *J* = 16.0 Hz, H-2′), 7.22 (1H, s, H-5′), 7.29 (1H, d, *J* = 15.0 Hz, H-3), and 7.31 (1H, d, *J* = 16.0 Hz, H-3′) (Table 2). Analysis of ^13^C NMR and HSQC data indicated 28 carbon signals attributable to two methyl groups at δ_C_ 18.4 (C-12′) and 23.8 (C-12); two methylenes at δ_C_ 23.5, (C-14) and 27.7 (C-14′); one aliphatic methine at δ_C_ 45.9 (C-8); two oxygenated methines at δ_C_ 69.0 (C-11) and 84.7 (C-7′); 13 olefinic methines at δ_C_ 120.9 (C-2), 122.8 (C-8′), 123.7 (C-2′), 128.0 (C-9), 131.6 (C-10′), 134.0 (C-11′), 136.8 (C-7), 139.1 (C-9′), 139.9 (C-3′), 141.0 (C-5), 141.1 (C-10), 145.3 (C-3), and 155.5 (C-5′); and eight nonprotonated carbons at δ_C_ 43.0 (C-6′), 128.8 (C-4′), 134.0 (C-4), 136.9 (C-6), 164.3 (C-13′), 170.1 (C-1), 170.1 (C-1′), and 172.2 (C-13). The COSY correlations (Figure S14) of H-2/H-3, H-7/H-8, H-8/H-9, H-9/H-10, H-10/H-11, and H-11/H_3_-12, together with key HMBC correlations (Figure S15) of H-2/C-1 and C-4; H-3/C-1, C-4, and C-5; H-5/C-4, C-6, and C-7; H-7/C-6, H-10/C-8; and H_3_-12/C-10, established the sequential connectivity from C-1 to C-12 carbon chain. Additional HMBC correlations from H-3 to C-13 and H-5 to C-13 allowed the placement of C-13 within this framework. Similarly, the COSY correlations of H-2′/H-3′, H-7′/H-8′, H-8′/H-9′, H-9′/H-10′, H-10′/H-11′, and H-11′/H_3_-12′, in combination with HMBC correlations from H-2′ to C-1′ and C-4′; H-3′ to C-1′ and C-4′; H-5′ to C-3′, C-4′, C-6′, and C-7′; H-8′ to C-6′; H-9′ to C-7′; and H-11′ to C-9′, indicated the connectivity from C-1′ to C-12′ carbon chain. Further COSY correlations of H-7/H-8 and H_2_-14/H_2_-14′, accompanied by HMBC correlations of H-7 to C-6; H-8 to C-5′; H_2_-14′ to C-6′; H-5′ to C-4′, C-6′, C-13′, and C-14′; H-7′ to C-5′, C-6′, and C-13′, established a scaffold of compound **2** consisting a cyclohexene ring and a six-membered α,β-unsaturated cyclic ketone sharing a spiro quaternary carbon. The configurations of Δ^2^, Δ^9^, Δ^2′^, Δ^8′^, and Δ^10′^ double bonds were deduced as *E* based on the coupling constants of *J*_H‑2/H‑3_ (15.0 Hz), *J*_H‑9/H‑10_ (15.3 Hz), *J*_H‑2′/H‑3′_ (16.0 Hz), *J*_H‑8′/H‑9′_ (15.0 Hz), and *J*_H‑10′/H‑11′_ (15.0 Hz), respectively. The NOESY cross-peak (Figure S16) observed between H-5′ and H-9 indicates that the substituents at C-6′ and C-8 are oriented on the same face (6′S*,8′S*). Likewise, the NOESY correlation between H-5′ and H-7′ suggests that the substituents at C-6′ and C-7′share the same-face orientation (6′S*,7′S*). The absolute configuration was subsequently investigated by comparing the experimental ECD spectrum with calculated spectra (see Figure 3). The experimental ECD spectrum of compound **2** exhibited a positive Cotton effect at 215 (θ–11.9 mdeg) and a negative Cotton effect at 228 nm (θ −37.5 mdeg). The calculated ECD spectrum of the 6′*S*,7′*S*,8*S*,11*R* stereoisomer, displaying a positive Cotton effect around 220 nm and a negative Cotton effect near 251 nm, showed good agreement with the experimental spectrum in both the sign and shape of the Cotton effects. However, a similar ECD profile was also observed for the calculated spectrum of the 6′*S*,7′*S*,8*S*,11*S* stereoisomer, indicating that the absolute configuration at C-11 could not be unambiguously established by ECD analysis alone. Accordingly, the absolute configurations at C-6′, C-7′, and C-8 were assigned as *S* (Figure 2B).

The spectroscopic data of compound **3** were very similar to those of compound **2** except that H-8 at δ_H_ 3.13 in **2** shifted to δ_H_ 3.04 in **3**; H_2_-14 at δ_H_ 2.26 and 2.37 in **2** shifted to δ_H_ 2.40 in **3**; H-5′ at δ_H_ 7.22 in **2** shifted to δ_H_ 7.00 in **3**; and H_2_-14′ at δ_H_ 1.76 and 1.98 in **2** shifted to δ_H_ 1.76 and 1.82 in **3** in the ^1^H NMR spectrum; and C-8 at δ_C_ 45.9 in **2** shifted to δ_C_ 49.3 in **3** in the ^13^C NMR spectrum. Further complete 2D NMR analysis (Figures 1 and S21–S23) confirmed that compound **3** possessed the same planar structure as that of compound **2**, suggesting they were diastereoisomers. The geometry of the Δ^2^, Δ^9^, Δ^2^′, Δ^8^′, and Δ^10^′ in **3** were determined to be the same as those of **2** due to similar *J* value patterns between **3** and **2**. In contrast to compound **2**, a distinctive NOESY cross-peak (Figure S24) between H-5′ and H-8 was observed for compound **3**, whereas no NOESY correlation was detected between H-5′ and H-9. This observation suggested that the substituents at C-6′ and C-8 were located on opposite faces. The NOESY cross-peak between H-7′ and H-14 supported a same-face orientation of the substituents at C-7′ and C-6′. Owing to the overlap of the NOESY cross-peaks of H-11/H-10 and H-9/H-10, the relative configuration of C-11 could not be directly judged from the interproton distance calculation. The experimental ECD spectrum of compound **3** exhibited a positive Cotton effect at 212 nm (θ + 51.9 mdeg) and a negative Cotton effect at 231 nm (θ–113.5 mdeg). The calculated ECD spectrum of the 6′*R*,7′*S*,8*S*,11*R* stereoisomer, displaying a positive Cotton effect at 218 nm and a negative one near 249 nm, was closely matched with the experimental curve in both the sign and overall shape of the Cotton effects. However, a very similar ECD profile was also observed for the calculated spectrum of the 6′*R*,7′*S*,8*S*,11*S* epimer, indicating that the absolute configuration of C-11 could not be determined using ECD analysis. Thus, the absolute configurations of C-6′, C-7′, and C-8 were assigned as *R*, *S*, and *S*, respectively.

To determine the stereochemistry of compounds **1**–**3**, attempts were made to obtain single crystals suitable for X-ray diffraction analysis. However, all crystallization trials were unsuccessful.

Cell viability of compounds **1–7** and curcumin (Cur) and paclitaxel (PTX) in BV-2 microglial cells (Figure 4A) and the effects of compounds **1**–**7** on NO production in LPS-stimulated BV-2 cells were examined at 20 μM (Figure 4B). Moreover, the IC_50_ values were determined (Table 3), with compounds **1**, **2**, and **3** showing potent inhibition (11.1 ± 0.5 and 9.9 ± 0.1 μM, respectively), which were slightly weaker than that of the positive control curcumin (IC_50_ = 2.7 ± 0.3 μM). Compounds **4**–**7** exhibited moderate NO inhibitory effects, although the effects of compounds **4** and **6** may be partially due to cytotoxicity.

**Figure 4:**
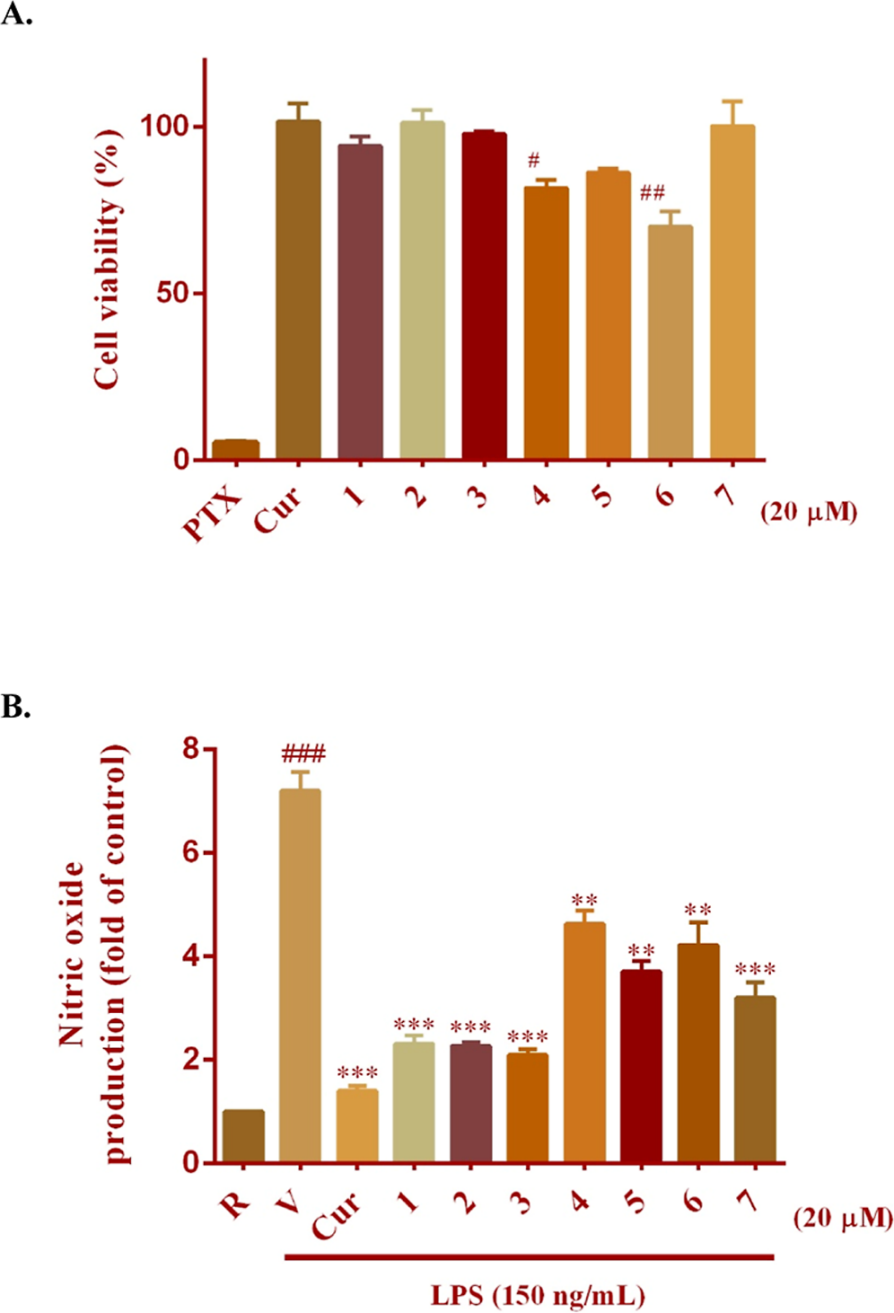
Cell viability of compounds **1–7**, curcumin (Cur), and paclitaxel (PTX) in BV-2 microglial cells (A) and their effects on LPS-induced NO production (B). The concentration of test compounds was 20 μM. Data are expressed as the mean ± SD (*n* = 3). ^#^ *p* < 0.05 and ^###^ *p* < 0.001, compared with the resting group (R); *p < 0.05, **p < 0.01 and ***p < 0.001, compared with the group of stimulation (V).

**Table 3:** IC_50_ Values of 1–3 on Nitric Oxide Production Inhibitory Activities Induced by Lipopolysaccharide in Microglial BV-2 Cells

compound	IC_50_ (μM)^a^	cell viability (%)
**1**	10.3 ± 0.5	92.9 ± 5.3
**2**	11.3 ± 0.2	102.2 ± 2.8
**3**	9.9 ± 0.6	101.5 ± 8.8
Curcumin^b^	2.7 ± 0.3	100.6 ± 6.0

a IC_50_ = concentration that reduces NO production by 50%.

b Positive control used in this study.

Western blot analysis demonstrated that treatment with compounds **2** and **3** (20 μM) significantly downregulated iNOS protein expression in LPS-stimulated BV-2 microglial cells (*p* < 0.001), whereas compound **1** exerted only a marginal inhibitory effect (Figure 5A and 5B). Curcumin, used as the positive control, exhibited the most pronounced suppression of iNOS expression. These results suggest that the observed NO inhibitory activity is closely associated with the suppression of iNOS protein expression, thereby supporting compounds **2** and **3** as potential iNOS inhibitors. This observation is consistent with previous reports identifying iNOS as a key therapeutic target in the modulation of neuroinflammatory responses.

**Figure 5:**
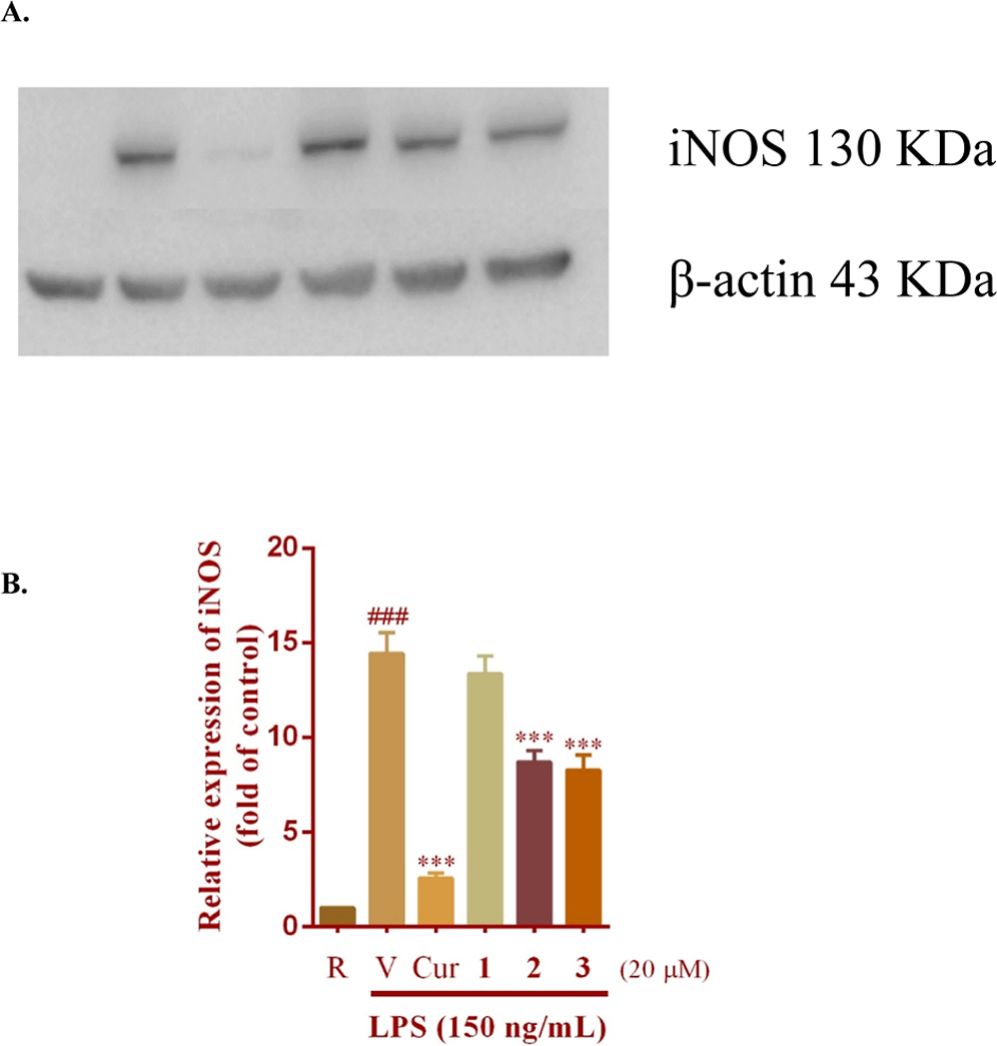
Effects of compounds 1–3 and curcumin (Cur) on LPS-induced iNOS expression (A,B). The concentration of test compounds was 20 μM. Data are expressed as the mean ± SD (*n* = 3). ^#^ *p* < 0.05 and ^###^ *p* < 0.001, compared with the resting group (R); *p < 0.05, **p < 0.01 and ***p < 0.001, compared with the group of stimulation (V).

Taken together, the NO inhibition assay and Western blot analysis consistently demonstrated that compounds **2** and **3** exerted more pronounced suppressive effects on LPS-induced NO production and iNOS protein expression in BV-2 microglial cells than compound **1**, while maintaining minimal cytotoxicity at the tested concentration. These results suggest that the observed inhibition of NO production is closely associated with the downregulation of iNOS expression and prompted further investigation into the potential molecular basis underlying the differential inhibitory activities of compounds **1**–**3**.

### Molecular Docking Analysis of Compounds 1–3 with iNOS

To gain mechanistic insight into the inhibitory effects of compounds **1**–**3** on inducible nitric oxide synthase (iNOS), molecular docking analysis was performed using the crystal structure of the murine iNOS heme domain (PDB ID: 1QW4), which has been widely used to characterize the catalytic core responsible for l-arginine recognition and nitric oxide biosynthesis.^19^

Representative three-dimensional binding conformations of compounds **1**–**3**, together with the reference inhibitor curcumin, are illustrated in Figure 6, and the corresponding interaction patterns are summarized in Table S1.

**Figure 6:**
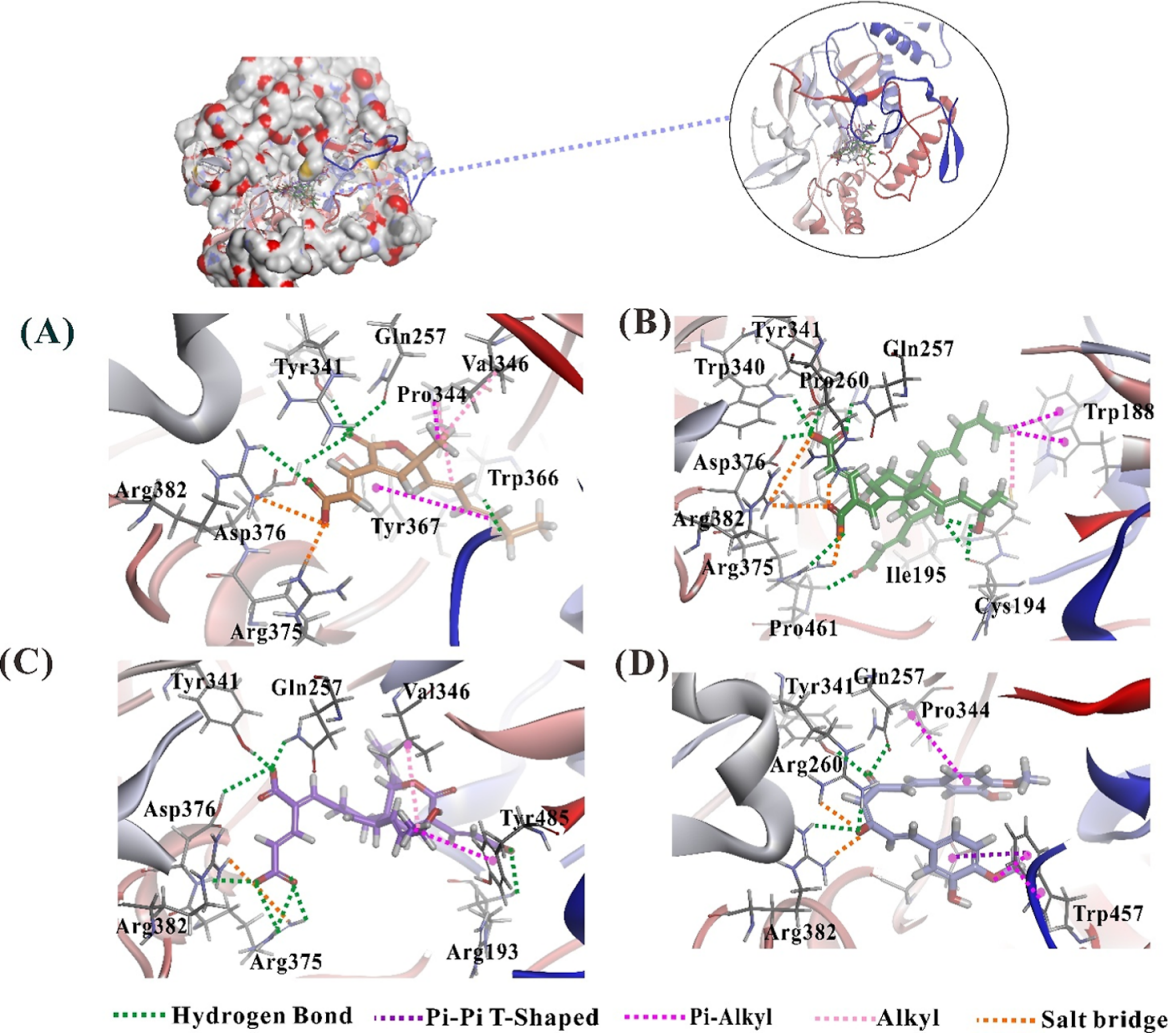
Three-dimensional docking models illustrating representative binding conformations of compound 1 (A), compound 2 (B), compound 3 (C), and curcumin (D) within the catalytic pocket of inducible nitric oxide synthase (iNOS; PDB ID: 1QW4). Key active-site residues are shown as stick representations. This figure highlights the interaction hierarchy and cooperative binding networks formed between the ligands and the iNOS active site. Hydrogen bonds are indicated by green dashed lines, Pi–Pi T-shaped interactions by purple dashed lines, Pi–alkyl interactions by magenta dashed lines, alkyl interactions by pink dashed lines, and salt bridge (attractive charge) interactions by orange dashed lines.

The docking results indicate that all tested compounds are capable of occupying the canonical and conserved l-arginine recognition pocket of iNOS, engaging residues known to be critical for substrate recognition and catalysis, including Arg260, Glu371, Asp376, and Tyr367, which are highly conserved between murine and human iNOS isoforms.^20^ Curcumin, used as a reference compound, exhibited extensive interactions with multiple active-site residues, forming a dense interaction network consistent with its reported iNOS Together, these results highlight the importance of hydrogen-bond networks inhibitory activity.^21^

Among the tested compounds, compounds **2** and **3** displayed favorable and well-defined binding orientations within the iNOS active site. As shown in Figure 6, both compounds adopted conformations that enabled simultaneous interactions with key catalytic residues, including Arg260, Glu371, Asp376, Tyr367, and Arg382. These interactions consisted of a combination of hydrogen bonding, electrostatic contacts (salt bridge/attractive charge), and hydrophobic interactions, forming cooperative interaction networks that stabilize the ligands within the catalytic cavity.

Although compounds **2** and **3** exhibited comparable inhibitory effects on iNOS expression in the Western blot analysis, their docking poses suggested distinct yet similarly effective binding modes. Compound **3** adopted a more compact binding orientation, characterized by clustered interactions around the catalytic core residues, whereas compound **2** engaged the active site through a more distributed interaction pattern involving both polar and hydrophobic regions of the binding pocket. Such differences in interaction topology may contribute to their similar biological activities despite differences in molecular orientation and binding geometry.

In contrast, compound **1** displayed a less optimized binding orientation, characterized by fewer cooperative interactions with the core catalytic residues of iNOS. The reduced interaction density and limited stabilization within the active site are consistent with its relatively weaker inhibitory effect observed experimentally.

The combined biological and molecular docking results suggest that effective suppression of iNOS activity by compounds **2** and **3** is not governed by a single dominant interaction, but rather by the formation of cooperative interaction networks within the catalytic pocket of iNOS. In particular, hydrogen-bond interactions involving Asp376, Glu371, and Tyr367, together with stabilizing salt bridge interactions with positively charged residues such as Arg260 and Arg382, appear to play a central role in anchoring the ligands in orientations favorable for functional inhibition.

Notably, salt bridge or attractive charge interactions were observed for all three compounds as well as for the reference compound curcumin, indicating that electrostatic complementarity alone is insufficient to account for differences in inhibitory potency. Instead, the presence and organization of hydrogen-bond networks, which provide directional and cooperative stabilization within the active site, appear to better correlate with the experimentally observed differences in NO inhibition and iNOS downregulation.

This interpretation is consistent with previous structural studies demonstrating that residues within the l-arginine binding pocket, including Arg260, Asp376, and Glu371, play critical roles in substrate recognition and inhibitor binding in iNOS. In our previous study, molecular docking analysis employing a similar CDOCKER-based protocol emphasized the relationship between estimated binding energies and experimental inhibition data. In the present work, docking analysis was instead used primarily to provide mechanistic insight into how structurally related compounds engage the canonical iNOS active site, thereby complementing the observed cellular and molecular inhibitory effects.

In summary, compounds **1**–**3** were evaluated for their inhibitory effects on nitric oxide production and iNOS expression in LPS-stimulated microglial cells. Among them, compounds **2** and **3** exhibited more pronounced suppression of NO production and iNOS protein expression compared to compound **1**. Molecular docking analysis using murine iNOS (PDB ID: 1QW4) provided structural insight into these observations, suggesting that effective inhibition is associated with the formation of cooperative interaction networks within the l-arginine binding pocket rather than a single dominant interaction. Together, these results highlight the importance of hydrogen-bond networks involving key catalytic residues in stabilizing ligand binding and support the potential of these compounds as structural templates for further development of iNOS-targeted inhibitors.

Furanone and γ-lactone-containing natural products are known to exhibit diverse biological activities, including anticancer, anti-inflammatory, and antimicrobial effects.^22,23^ Structure-activity relationship studies indicate that alkyl substitution at the C-5 position primarily modulates lipophilicity, whereas the incorporation of more functionalized substituents enhances biological activity.^22,24^ The presence of an α,β-unsaturated lactone moiety suggests potential Michael acceptor reactivity, which has been reported to modulate inflammatory signaling pathways through covalent interaction with nucleophilic residues.^24,25^ Additionally, multiple carboxylic acid groups may improve aqueous solubility but reduce membrane permeability, thereby limiting cytotoxic potency.^26,27^ These features are consistent with polyketide-derived natural products, where biological activity depends on functional group diversity and physicochemical balance.^23,28^

## Experimental Section

### General Experimental Procedures

Specific rotations were measured on a JASCO P-2000 Digital Polarimeter (JASCO, Tokyo, Japan). The UV spectra were recorded on a Thermo UV–visible Heλios α Spectrophotometer (Thermo Scientific, Waltham, MA, USA). The IR spectra were obtained on a JASCO FT/IR 4100 spectrometer (JASCO, Tokyo, Japan). The NMR spectra were recorded at 600 and 150 MHz for ^1^H and ^13^C, respectively, on Bruker AVI 500 MHz FT-NMR spectrometer (Bruker BioSpin GmbH, Ettlingen, Germany). HRESIMS spectra were determined on a Q Exactive Plus Hybrid Quadrupole-Orbitrap Mass Spectrometer (Thermo Fisher Scientific, Bremen, Germany). HPLC separation was performed on a Hitachi HPLC system coupled with a Bischoff RI-8120 RI Detector (Bischoff, Leonberg, Germany) and the Phenomenex Luna column (5 μm PFP column, 100 Å, 250 × 10.0 mm) (Torrance, CA, USA), SunFire column (5 μm C_18_ column, 100 Å, 250 × 10.0 mm) (Milford, Mass, USA) and Phenomenex Gemin column (5 μm C_18_ column, 110 Å, 250 × 4.6 mm) (Torrance, CA, USA). Open column chromatography was performed with Sephadex LH-20 (Amersham Bioscience, Uppsala, Sweden). TLC was carried out with precoated silica gel 60 F254 (Merck, Darmstadt, Germany). Compounds were detected by UV and 10% aqueous H_2_SO_4_ spraying reagent followed by heating at 105 °C for 1 min. The solvents were analytical grade MeOH (Merck, Darmstadt, Germany) for HPLC. ECD data were acquired with a J-815 Circular Dichroism Spectrometer (JASCO, Tokyo, Japan).

### Fungal Strain and Culture

The fungal strain of *W. dispersa* Ca4–13 was isolated from edible oysters *C. angulata* collected from Yunlin County, Taiwan. The strain was identified based on morphological characteristics and the molecular biology method by amplifying the D1R/D3Ca gene sequence. The sequence data for this strain have been deposited in GenBank with the accession number PQ005631. For cultivation, the purified strain was grown on a CMA medium plate (Becton, Dickinson and Company, Sparks, MD, USA) at 25 °C for 14 days. Agar cultures were cut into small pieces (approximately 0.5 × 0.5 × 0.5 cm^3^) and inoculated into one hundred and 50,250 mL flasks, each containing 100 mL medium (containing 2 g dextrose, 0.4 g peptone in 50 mL distilled water and 50 mL seawater) for liquid-state fermentation. The fermentation process was carried out at 25 °C for 30 days.

### Extraction and Isolation of Secondary Metabolites

The fermented broth (15.0 L) was filtered, then the filtrate was extracted with EtOAc (2 × 15.0 L), and the resulting extract was evaporated under reduced pressure to afford a viscous solid (2.9 g). The EtOAc extract was redissolved in 15 mL MeOH and subjected to Sephadex LH-20 column (2.5 i.d. × 64.0 cm), eluted with MeOH. The resulting fractions were combined into for groups (Fractions I–IV) based on their TLC analysis. Subsequently, fraction III further purified by semipreparative HPLC using phenomenex Luna PFP column with 60% MeOH/H_2_O containing 0.1% formic acid (2.0 mL/min) to obtain **1** (4.9 mg, *t*_R_ = 14.1 min), **2** (5.5 mg, *t*_R_ = 23.5 min) and **3** (10.3 mg, *t*_R_ = 26.1 min).

#### Westeroic Acid A (**1**)

Pale yellow powder; [α]­25 D −35.0 (*c* 0.1, MeOH); UV (MeOH) λmax (log ε) 228 (4.18) nm; HRESIMS *m*/*z* 263.0914 [M – H]^−^ (calcd 263.0924 for C_14_H_15_O_5_) (Figure S1); (ZnSe) *v*_max_: 3380, 2979, 2927, 1748, 1699, 1649, 1377, 1344, 1246, 1191, 1135, 1107, 1052, 985, 941, 865 cm^–1^ (Figure S2); ^1^H NMR data (MeOH-*d*_*4*_, 600 MHz); ^13^C NMR data (MeOH-*d*_*4*_, 150 MHz) see Table 1.

### Westeroic Acid B (**2**)

Yellow powder; [α]­25 D −44.2 (*c* 0.1, MeOH); UV (MeOH) λmax (log ε) 230 (4.48) nm; HRESIMS *m*/*z* 509.1817 [M – H]^−^ (calcd 509.1817 for C_28_H_29_O_9_) (Figure S9); IR (ZnSe) *v*_max_: 3344, 2943, 2835, 2360, 2337, 1709, 1662, 1454, 1408, 1111, 1018 cm^–1^ (Figure S10); ^1^H NMR data (MeOH-*d*_*4*_, 600 MHz); ^13^C NMR data (MeOH-*d*_*4*_, 150 MHz) see Table 1.

### Westeroic Acid C (**3**)

Yellow powder; [α]­25 D + 17.3 (*c* 0.1, MeOH); UV (MeOH) λmax (log ε) 231 (4.40) nm; HRESIMS *m*/*z* 509.1798 [M – H]^−^ (calcd 509.1817 for C_28_H_29_O_9_) (Figure S1); IR (ZnSe) *v*_max_: 3415, 2927, 1706, 1631, 1435, 1389, 1276, 1214, 1184, 1136, 983, 869 cm^–1^ (Figure S18); ^1^H NMR data (MeOH-*d*_*4*_, 600 MHz); ^13^C NMR data (MeOH-*d*_*4*_, 150 MHz) see Table 1.

### Electric Circular Dichroism Calculation

The relative configiurations of compounds **1**–**3** were deduced from their NOESY spectra and ChemBio3D modeling. The configurations were reoptimized at the CAM-B3LYP/6–31G­(d) level by the density functional theory (DFT) method in the Gaussian 16 software (Gaussian, Inc., Wallingford, CT, USA).^29^ The theoretical calculations of ECD were performed using time-dependent DFT at the CAM-B3LYP/6–31G­(d) level in MeOH with the polarizable continuum model.^30^

### Cell Culture

The mouse microglial BV-2 cell line was cultured as described previously.^31^ Before experiments, cells were changed to 0.5% FBS media. Thereafter, cells were treated with vehicle or the indicated concentration of compounds **1**–**7** for 30 min and then stimulated with LPS (L2880, Sigma-Aldrich, St. Louis, US) (150 ng/mL) for 24 h.

### Cell Viability Assay

The cell viability of compounds addressed in this study against the mouse microglial BV-2 cell line. The cell viability studies were determined by the MTT ([3-(4,5-dimethylthiazol-2-yl)-2,5-diphenyltetrazolium bromide, Sigma-Aldrich, St. Louis, US) method. Cells were seeded in 24-well plates at 5 × 10^5^ cells per well and grown for 24 h before use. The seeded cells were first treated with test compounds at 20 μM for 24 h. The final concentration of DMSO in the culture medium of the treated cells was adjusted to less than 0.5% (v/v) to prevent a solvent effect. Absorbance at 550 nm was obtained by a microplate reader (MRX). All of the experiments were performed in triplicate.^32^

### Inhibitory Activity of Nitric Oxide (NO) Production

Production of NO was evaluated by measuring the levels of nitrite in a conditioned medium as previously described with some modification.^33^ The culture supernatants were allowed to react with reconstituted cofactor solution and reconstituted nitrate reductase solution for 1 h at room temperature in the dark according to the instructions of the Nitrate/Nitrite Colorimetric Assay Kit (Cayman). Absorptions were measured at 550 nm using a microplate reader (MRX). Nitrite concentrations were calculated from the standard solutions of sodium nitrite. Curcumin was used as a positive control.^34^

### Western Blot Analysis

Western blot analysis was performed as previously described.^35^ Briefly, BV-2 cells were cultured as 80% confluence and changed to serum-free medium for 24 h. Thereafter, cells were treated with DMSO or the isolated compound, then stimulated with LPS (150 ng/mL) for 24 h. The quantitative supernatants from cellular lysates were subjected to SDS-PAGE and electrophoretically transferred onto a polyvinylidene fluoride membrane. After soak in the blocking buffer with 5% dry skim milk overnight, membranes were washed three times and sequentially incubated with primary antibodies (anti-iNOS) and HRP-conjugated secondary antibodies, followed by enhanced chemiluminescence detection. Data of specific protein levels are presented as the relative multiples in relation to the control groups.

### Molecular Dockings Analysis

Molecular docking analysis was performed using the CDOCKER protocol implemented in Discovery Studio 2021 (Dassault Systèmes BIOVIA, San Diego, CA, USA) to examine the binding modes of selected compounds within inducible nitric oxide synthase (iNOS). The crystal structure of murine iNOS (PDB ID: 1QW4) was obtained from the Protein Data Bank.

The chemical structures of compounds **1**–**3** and the reference compound curcumin were generated and converted into three-dimensional conformations, followed by energy minimization using the CHARMm force field in Discovery Studio. The protein structure was prepared using standard procedures, including removal of crystallographic water molecules, addition of hydrogen atoms, and optimization of protonation states prior to docking.

The binding site was identified based on PDB annotations and further refined using the Binding Site module in Discovery Studio. A spherical binding region was defined to encompass the l-arginine recognition pocket (center coordinates: *x* = −51.1075, *y* = 133.485, *z* = 47.2103; radius = 18 Å) and was used to guide ligand placement.

Energy-minimized ligand structures were docked into the defined active site using the CDOCKER algorithm. Representative low-energy binding conformations were selected for subsequent interaction analysis. The selected docking poses were used to qualitatively assess ligand–protein interaction patterns and to provide structural insight into the experimentally observed inhibitory effects.^10,36^

### Statistical Analysis

All experiments were performed in triplicate (*n* = 3) and the data are presented as means ± standard deviations (SD). Statistical analysis was conducted using the GraphPad Prism version. 9.5.1 (GraphPad Software, San Diego, CA, USA). One-way analysis of variance (ANOVA) followed by Tukey’s post hoc test was used for group comparisons.^37^ Statistical significance was determined based on the difference between groups, with *p* ≤ 0.05 considered statistically significant.

## Supplementary Material











## Data Availability

The data underlying this study have been deposited in Harvard Dataverse and are publicly available at 10.7910/DVN/M7OSG8.

## Notes

The raw NMR data for compounds **1**–**3** are also available in the (FID files), as well as the GenBank sequences of the fungal strain (ZIP)
compound1NMRraw.zip (ZIP)
compound2NMRraw.zip (ZIP)
compound3NMRraw.zip (ZIP)
Additional information, including HRMS, IR, and NMR spectra for compounds **1**–**3**; ^1^H NMR spectra for compounds **4**–**7**; and detailed tables of molecular docking results (PDF)
