# Supplementary material for: Spiro-Linked Polyketides from Cultures of Westerdykella dispersa Ca4–13 as Inhibitors of iNOS-Associated Neuroinflammation
Source: J Nat Prod. 2026 May 28;89(6):1775–84. doi: 10.1021/acs.jnatprod.6c00217 (PMC13316987; doi:10.1021/acs.jnatprod.6c00217)
Supplement: Supplementary file 5 [file np6c00217_si_005.pdf]

Supporting Information

Spiro-linked Polyketides from Cultures of Westerdykella dispersa Ca4-13 as Inhibitors of iNOS-Associated Neuroinflammation

Shu-Jung Huang [a], *Su-Jung Hsu* [a], *Yi-Chien Liu* [b, c], *Cheng-Yan Jiang* [b, c],
George Hsiao [b, c, d] *, *Tzong-Huei Lee* [a, e] *

[a] *Institute of Fisheries Science, National Taiwan University, Taipei 106319, Taiwan*
[b] *Ph.D. Program in Drug Discovery and Development Industry, College of Pharmacy, Taipei Medical University, Taipei 110301, Taiwan*
[c] *Department of Pharmacology, School of Medicine, College of Medicine, Taipei Medical University, Taipei 110301, Taiwan.*
[d] *Graduate Institute of Medical Sciences, College of Medicine, Taipei Medical University, Taipei 110301, Taiwan.*
[e] *Department of Life Science, College of Life Science, National Taiwan University, 106319, Taipei, Taiwan.*

Corresponding Author
Email: geohsiao@tmu.edu.tw (G.H.); thlee1@ntu.edu.tw (T.-H.L.)

Table of Contents

Figure S1. HRESIMS spectrum of compound **1**……….……………………..……..…....3
Figure S2. IR (ZnSe) spectrum of compound **1**……………………………..……….…..…3
Figure S3. ^{1}H NMR (600 MHz, methanol-d_4) spectrum of compound **1** ……………...4
Figure S4. ^{13}C NMR (150 MHz, methanol-d_4) spectrum of compound **1**……….……5
Figure S5. HSQC spectrum of compound **1**………………….………………………..…6
Figure S6. COSY spectrum of compound **1**………………… ………….…………7
Figure S7. HMBC spectrum of compound **1**………………. …………...………..8
Figure S8. NOESY spectrum of compound **1**………………. ………...…………9
Figure S9. HRESIMS spectrum of compound **2**………………. …………………10
Figure S10. IR (ZnSe) spectrum of compound **2...**……………. …………………10
Figure S11. ^{1}H NMR (600 MHz, methanol-d_4) spectrum of compound **2**…………...11
Figure S12. ^{13}C NMR (150 MHz, methanol-d_4) spectrum of compound **2**….……….12
Figure S13. HSQC spectrum of compound **2**…………………….…………………...13
Figure S14. COSY spectrum of compound **2**……………………………………....14
Figure S15. HMBC spectrum of compound **2**. ………………………………….…...15
Figure S16. NOESY spectrum of compound **2**. ……………………………………..16
Figure S17. HRESIMS spectrum of compound **3**. ………………….……………17
Figure S18. IR (ZnSe) spectrum of compound **3**. ………………………………….…..17
Figure S19. ^{1}H NMR (600 MHz, methanol-d_4) spectrum of compound **3**. ………....18
Figure S20. ^{13}C NMR (150 MHz, methanol-d_4) spectrum of compound **3**…………19
Figure S21. HSQC spectrum of compound **3**……………………………………..20
Figure S22. COSY spectrum of compound **3**……………………….……………..21
Figure S23. HMBC spectrum of compound **3**……………………………….….…22
Figure S24. NOESY spectrum of compound **3**……………………………………....23
Figure S25. ^{1}H NMR (600 MHz, pyridine-d_5) spectrum of compound **4**…..………....24
Figure S26. ^{1}H NMR (600 MHz, methanol-d_4) spectrum of compound **5**……….….25
Figure S27. ^{1}H NMR (600 MHz, methanol- d_4) spectrum of compound **6**……….….26
Figure S28. ^{1}H NMR (600 MHz, methanol- $d4$) spectrum of compound **7**…………27
Table S1. CDOCKER energy and interaction pattern of compounds **1**–**3** and curcumin docked into iNOS (PDB ID: 1QW4)…………………….……………...28

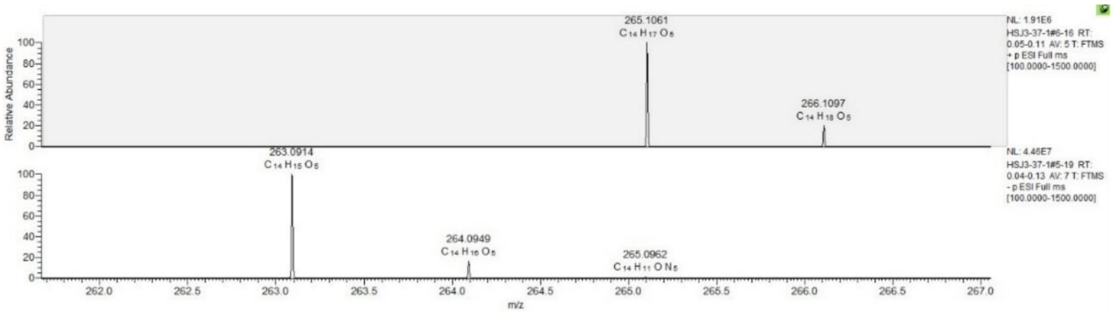

Figure S1. HRESIMS spectrum of compound **1**.

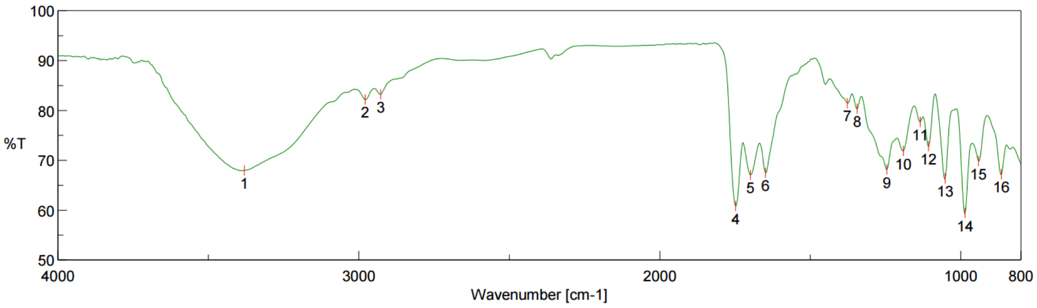

[Result of Peak Picking]

No.	Position	Intensity	No.	Position	Intensity	No.	Position	Intensity	No.	Position	Intensity	No.	Position	Intensity
1	3380.6	67.948	2	2979.48	82.1747	3	2927.41	83.2423	4	1748.16	60.8085	5	1698.98	67.0373
6	1648.84	67.515	7	1376.93	81.4497	8	1344.14	80.3128	9	1245.79	68.1387	10	1190.83	71.8723
11	1134.9	77.676	12	1106.94	72.7991	13	1051.98	66.3427	14	985.447	59.4361	15	941.092	69.8458
16	864.917	67.1869												

Figure S2. IR (ZnSe) spectrum of compound **1**.

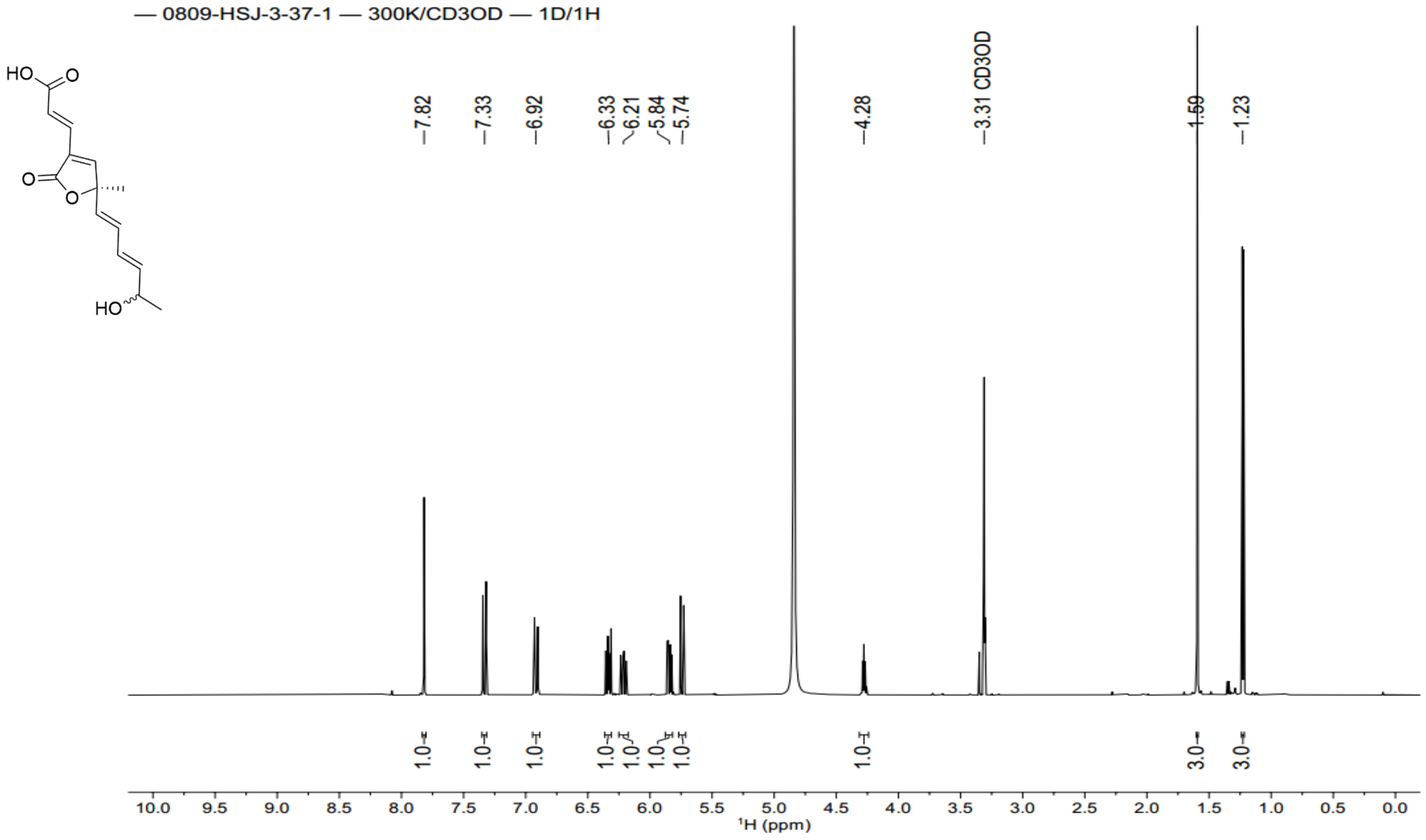

Figure S3. ^{1}H NMR (600 MHz, methanol-*d*$_4$) spectrum of compound **1**.

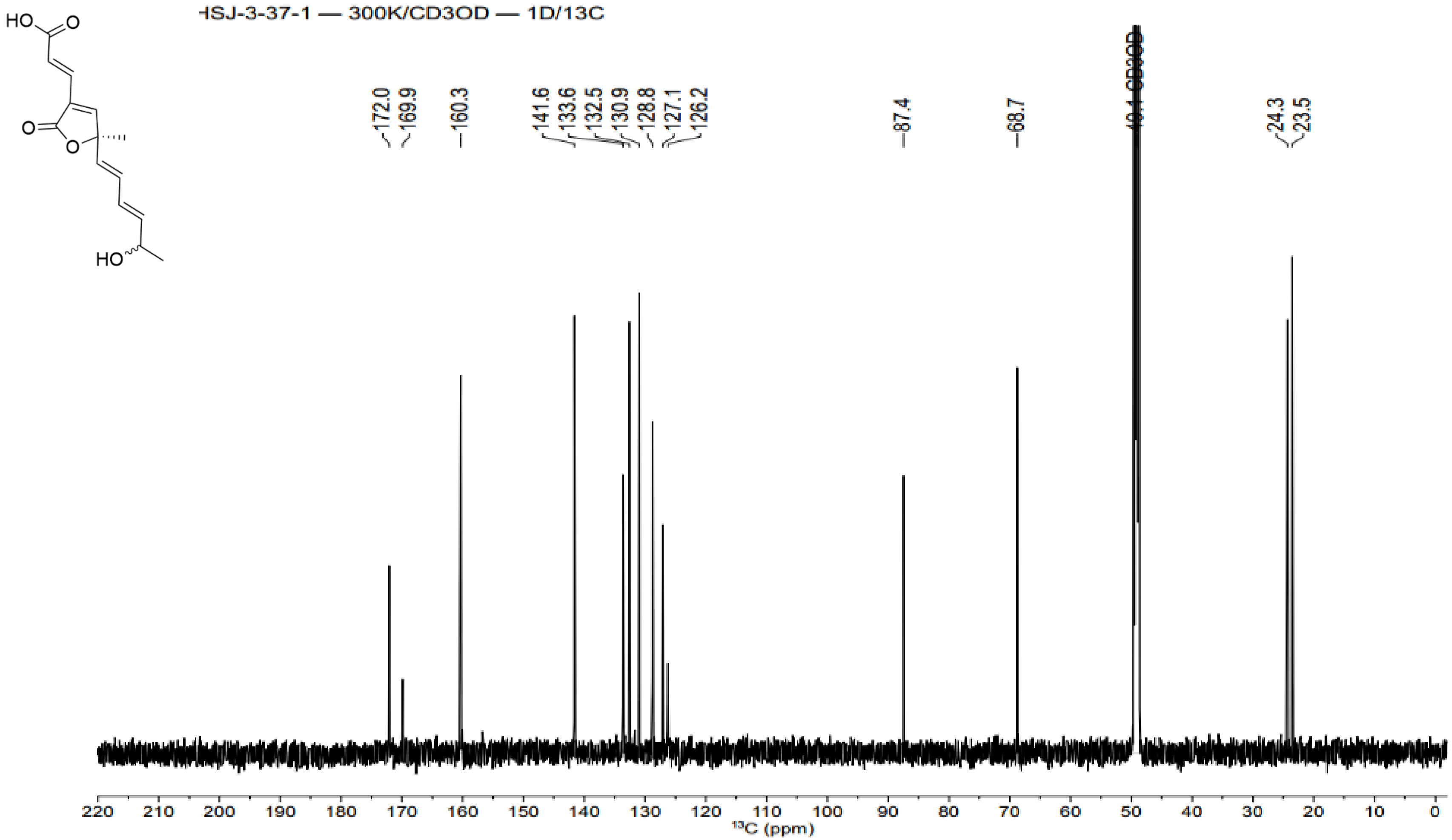

Figure S4. ^{13}C NMR (150 MHz, methanol-d_4) spectrum of compound **1**.

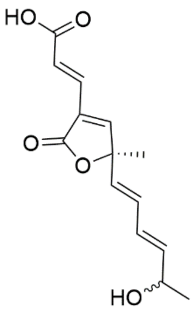

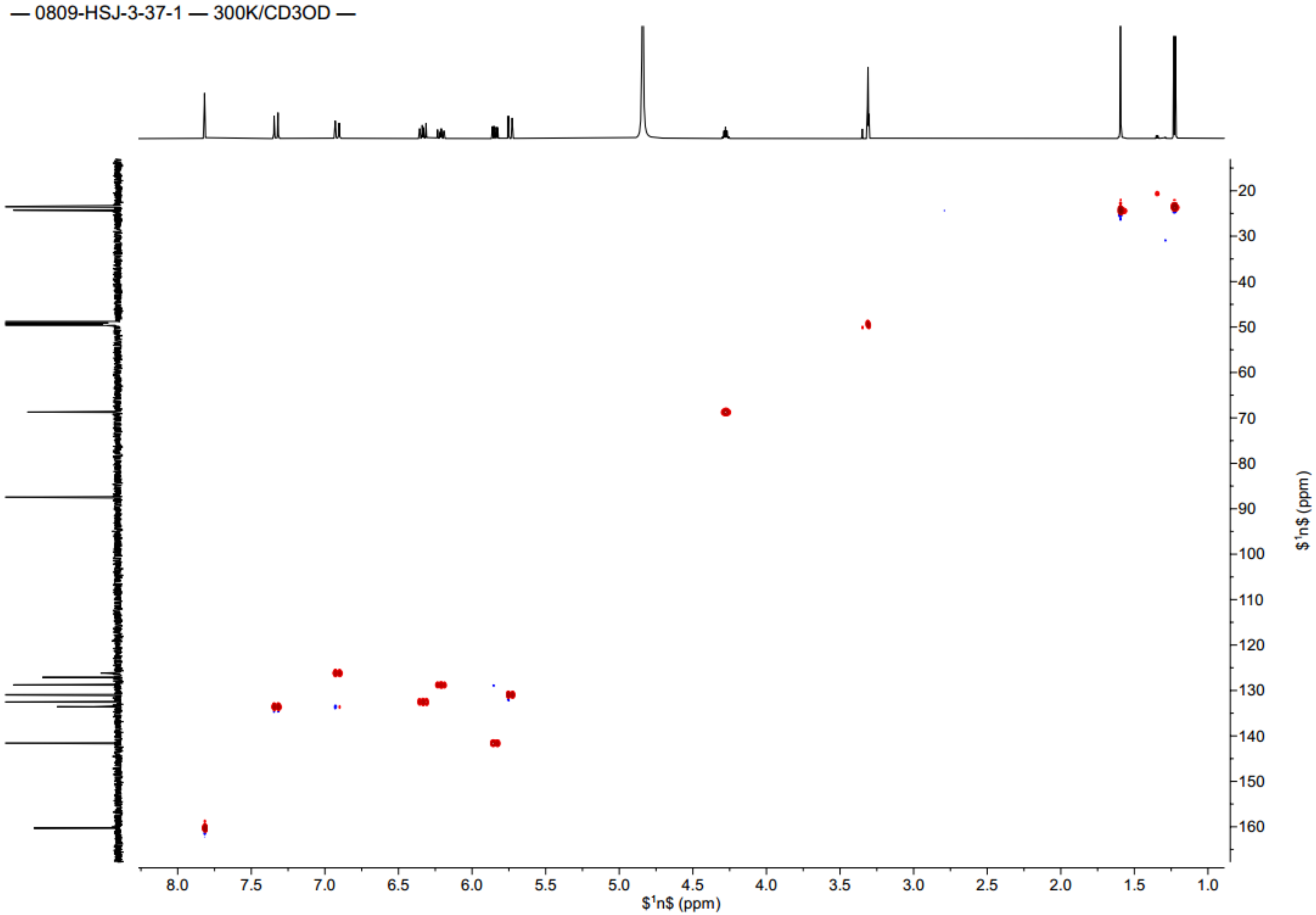

Figure S5. HSQC spectrum of compound **1**.

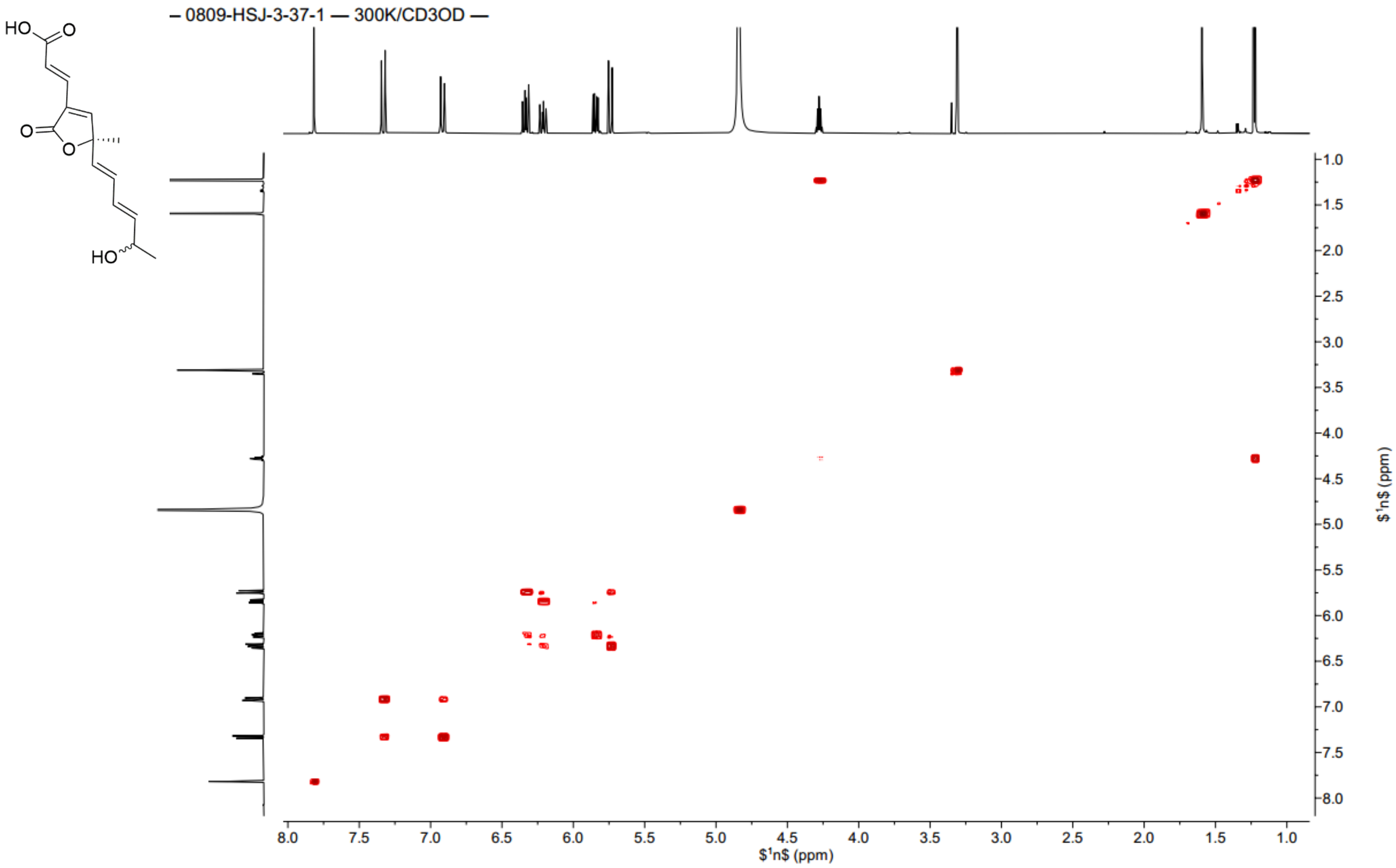

Figure S6. COSY spectrum of compound **1**.

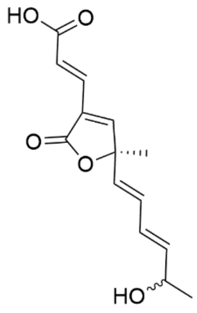

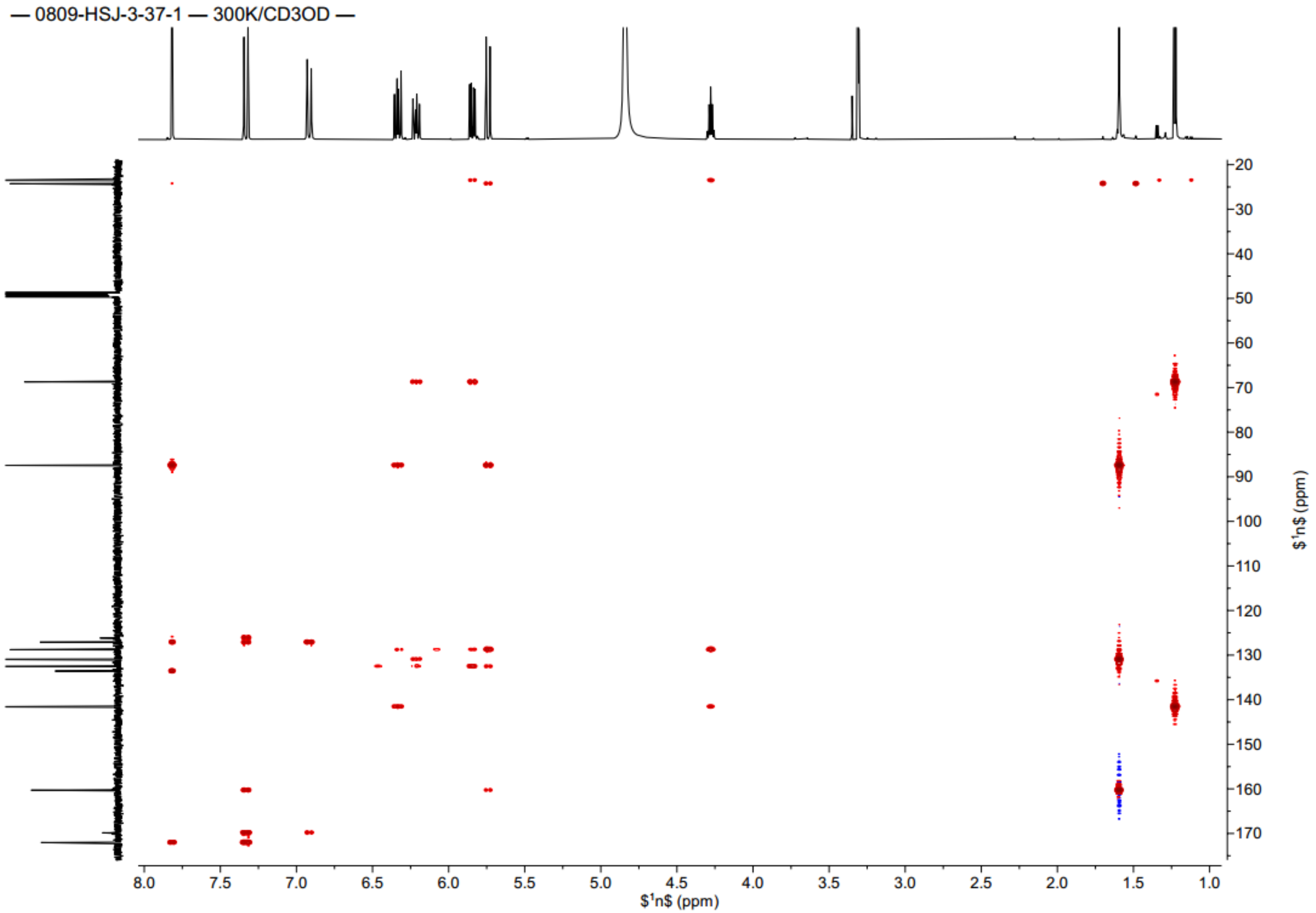

Figure S7. HMBC spectrum of compound **1**.

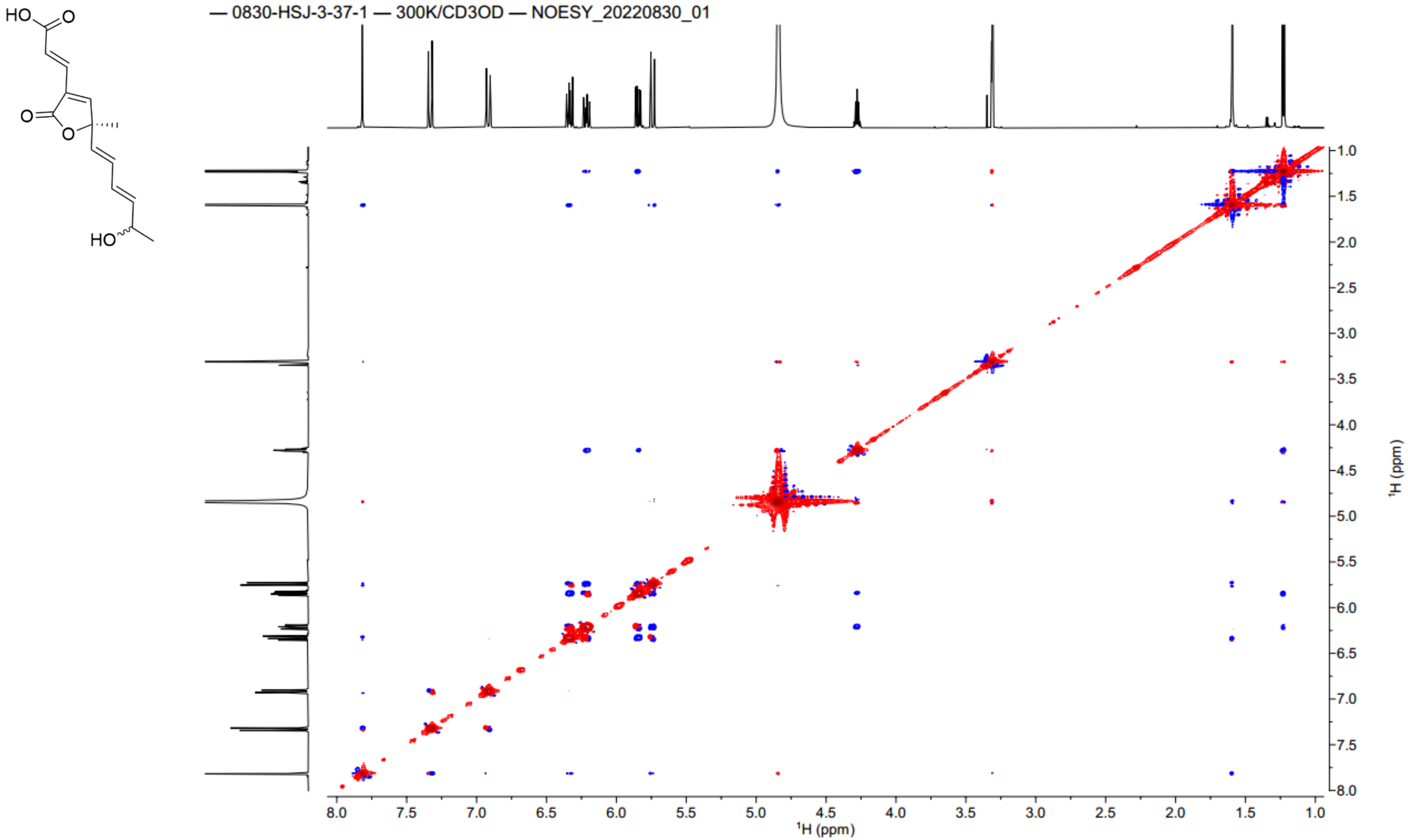

Figure S8. NOESY spectrum of compound **1**.

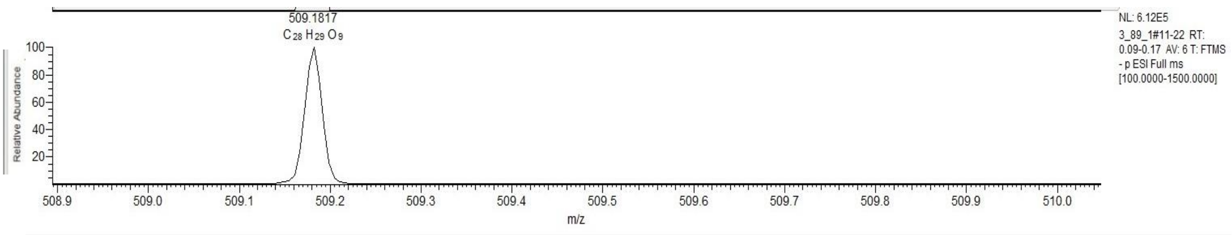

Figure S9. HRESIMS spectrum of compound **2**.

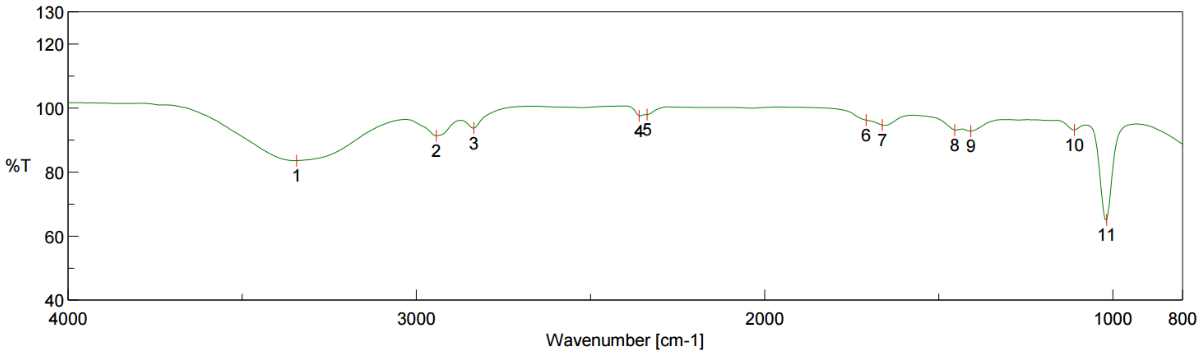

[Result of Peak Picking]

No.	Position	Intensity	No.	Position	Intensity	No.	Position	Intensity	No.	Position	Intensity	No.	Position	Intensity
1	3343.96	83.5313	2	2942.84	91.3071	3	2834.85	93.6695	4	2360.44	97.5006	5	2337.3	97.9143
6	1708.62	96.1902	7	1662.34	94.6676	8	1454.06	93.1018	9	1407.78	92.7705	10	1110.8	93.1363
11	1018.23	65.0565												

Figure S10. IR (ZnSe) spectrum of compound **2**.

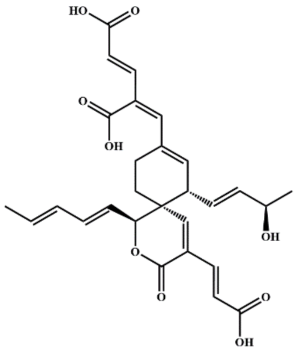

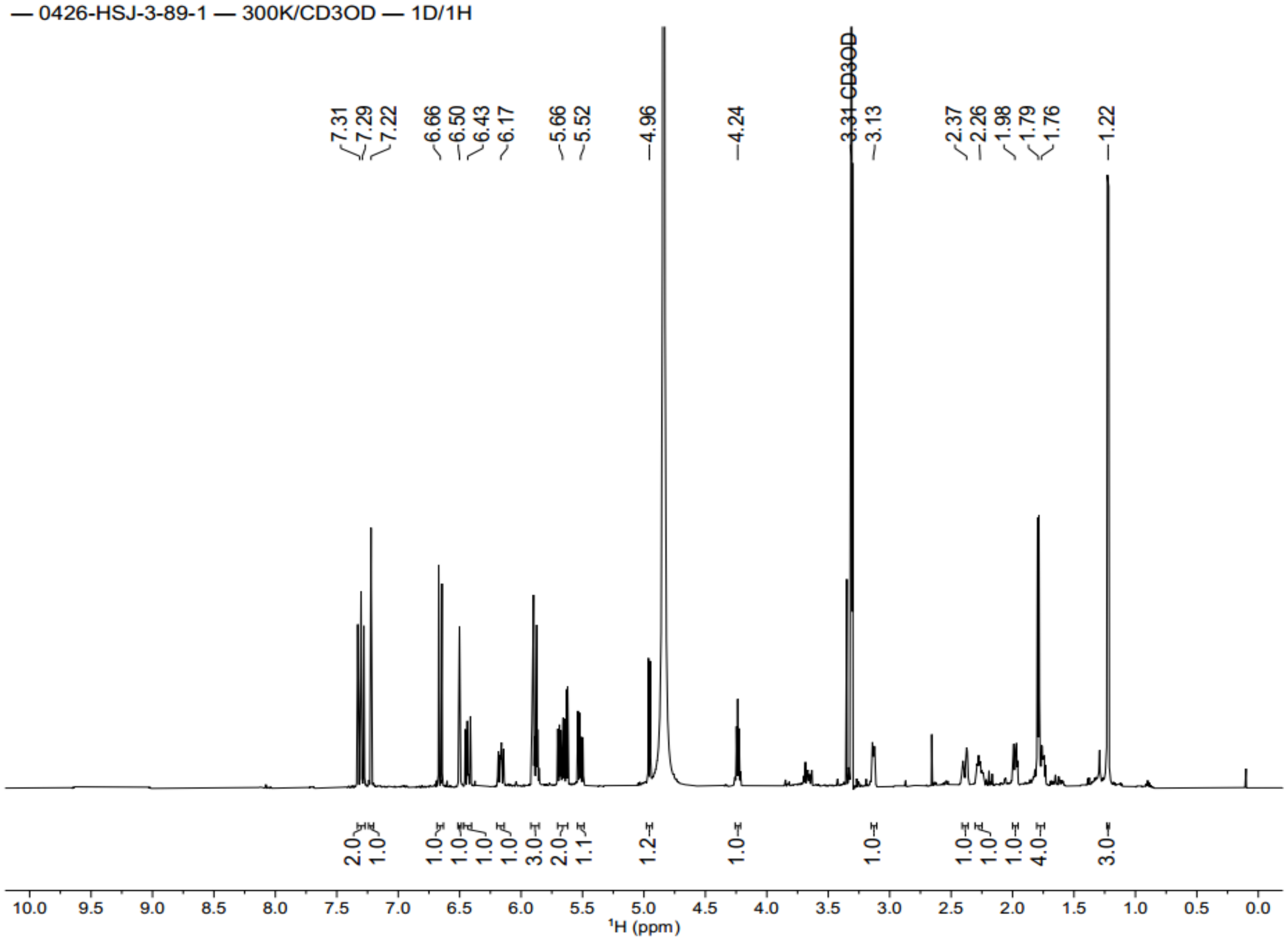

Figure S11. ^{1}H NMR (600 MHz, methanol-d_4) spectrum of compound **2**.

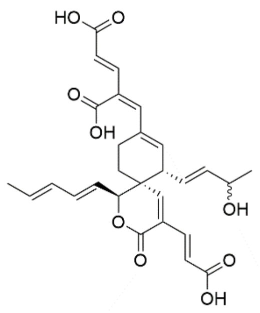

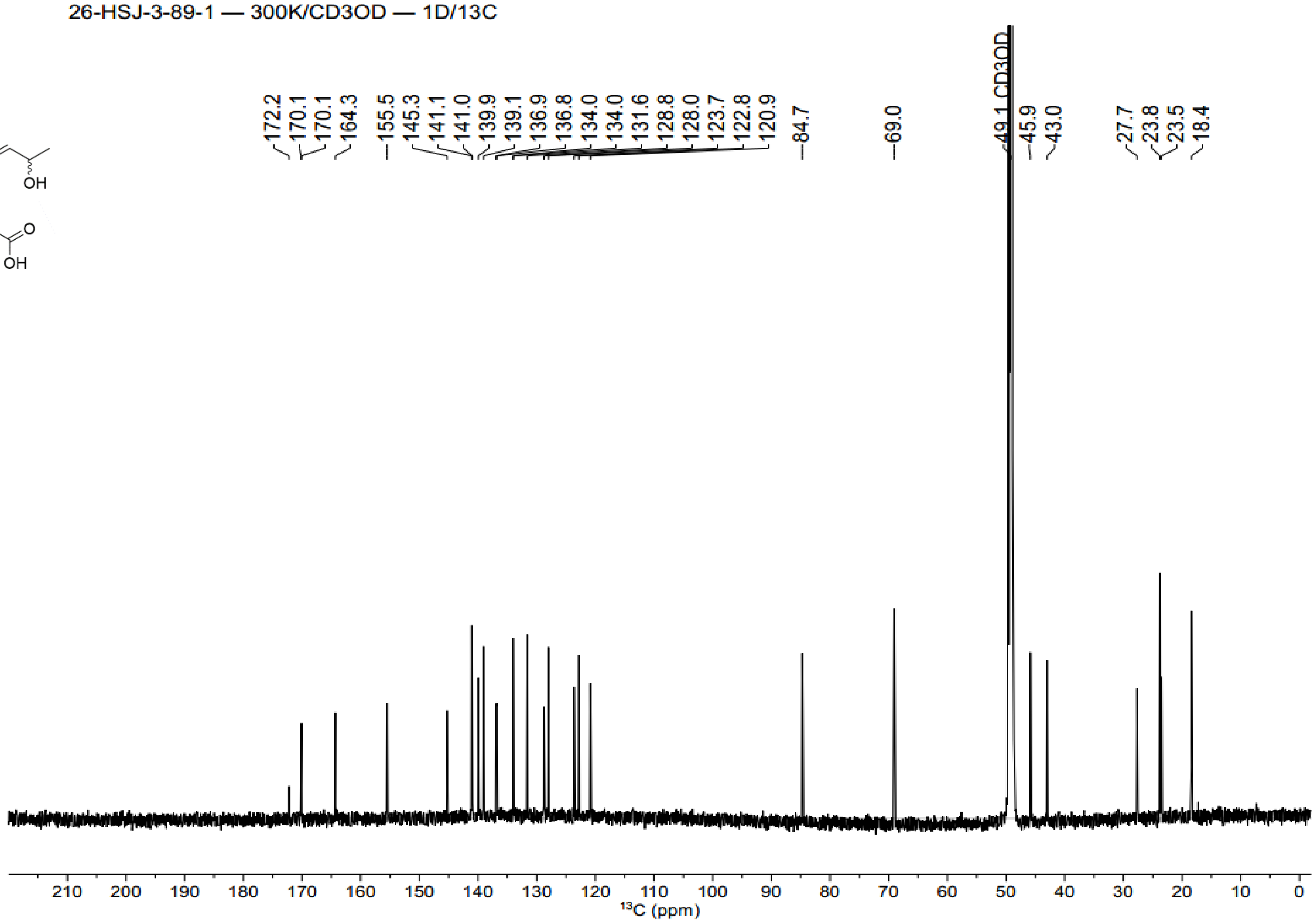

Figure S12. ^{13}C NMR (150 MHz, methanol-d_4) spectrum of compound **2**.

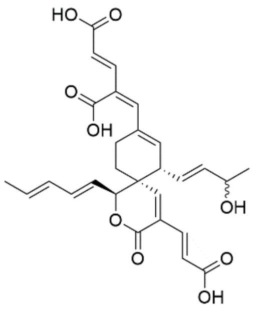

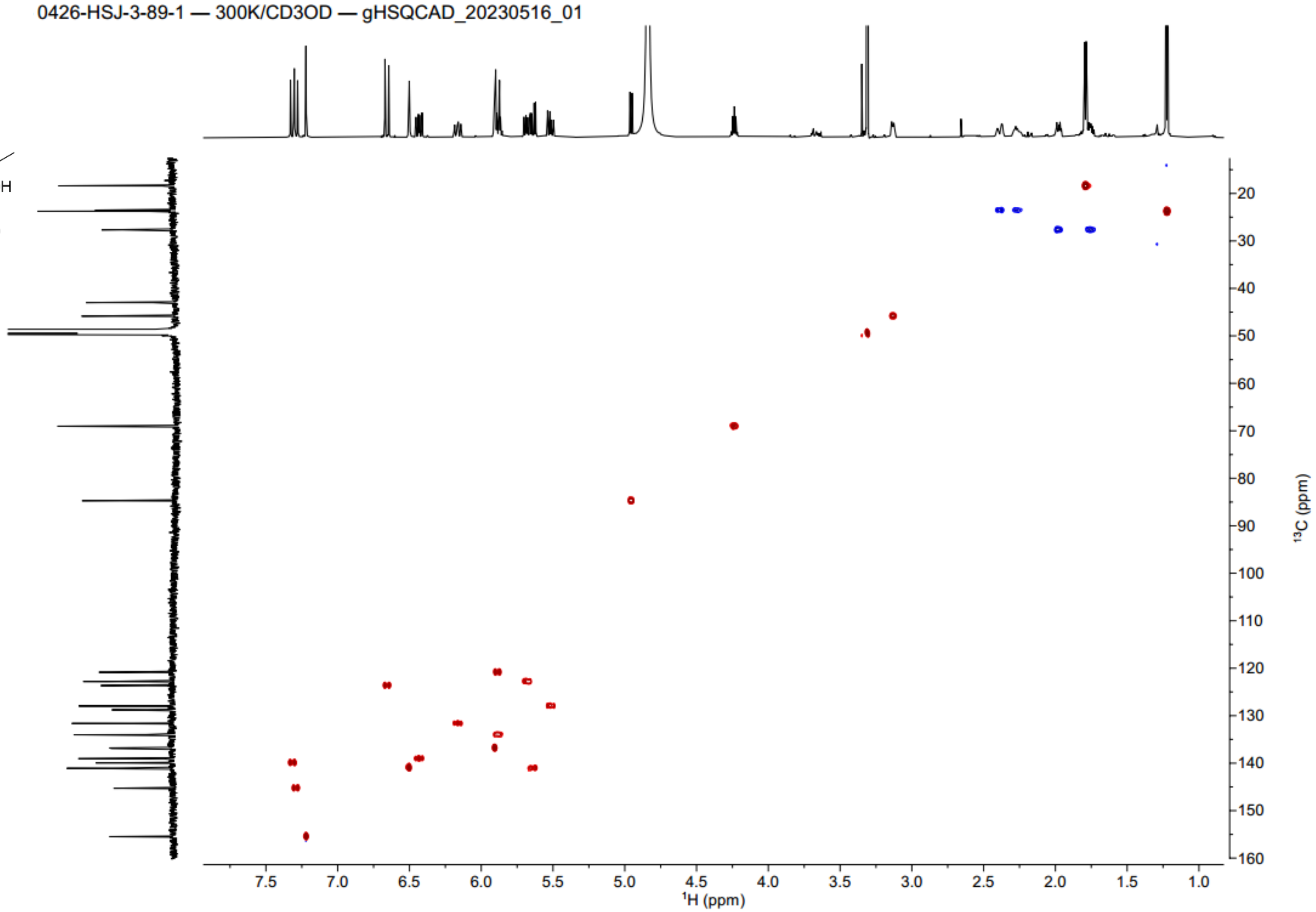

Figure S13. HSQC spectrum of compound **2**.

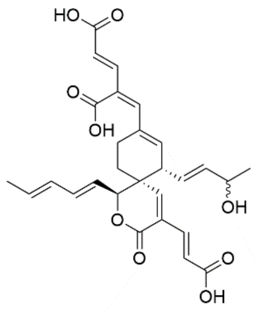

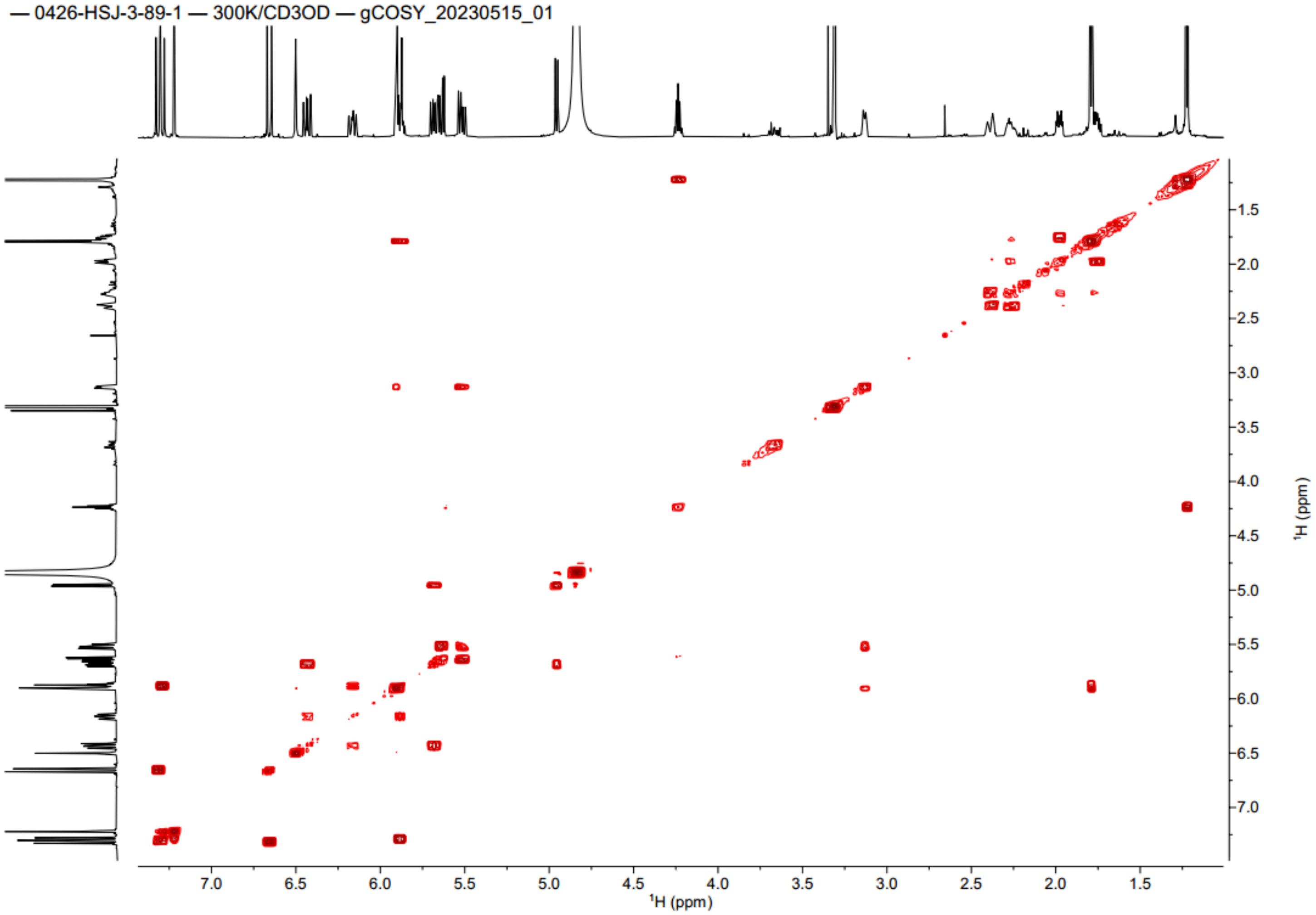

Figure S14. COSY spectrum of compound **2**.

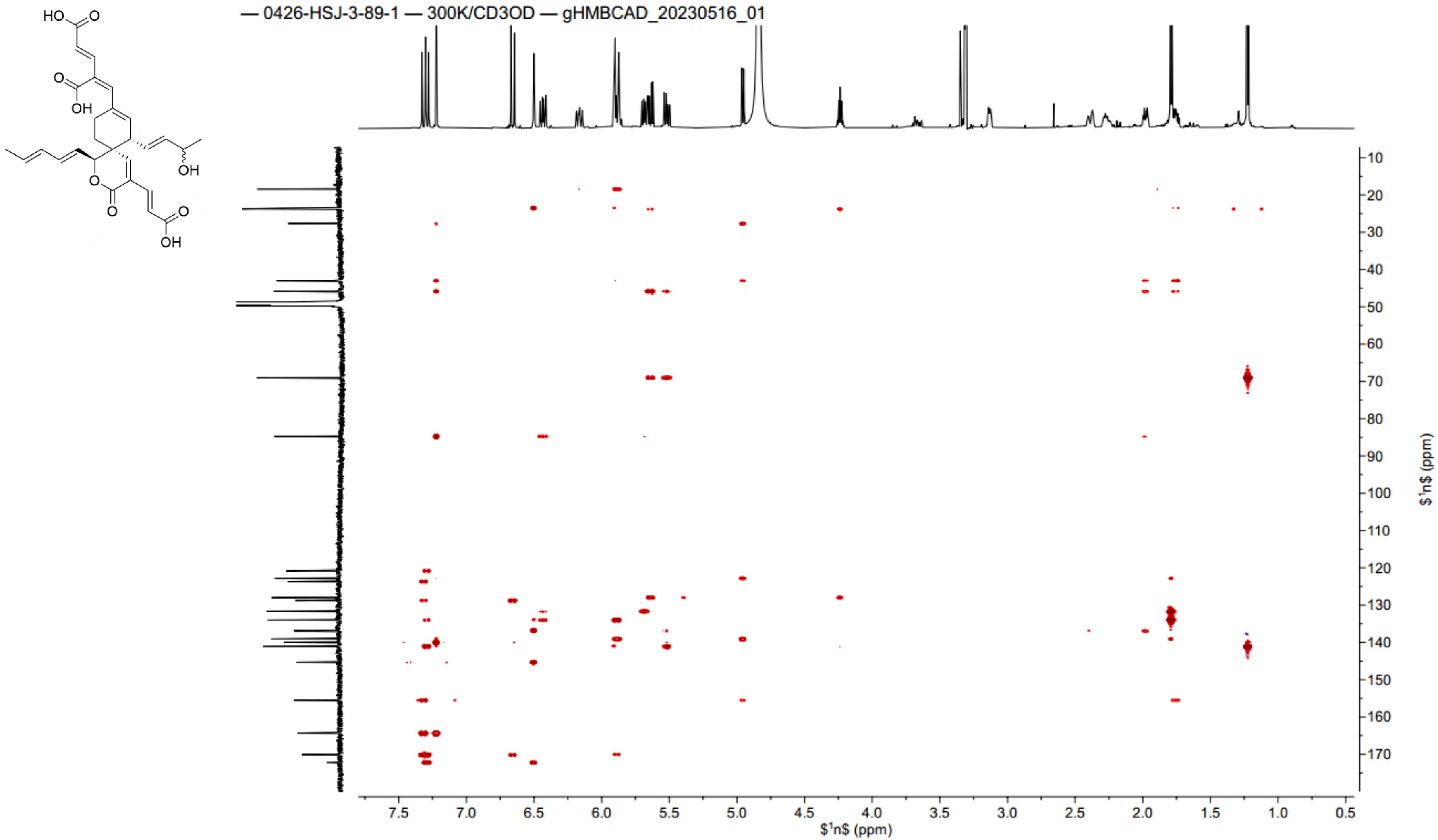

Figure S15. HMBC spectrum of compound **2**.

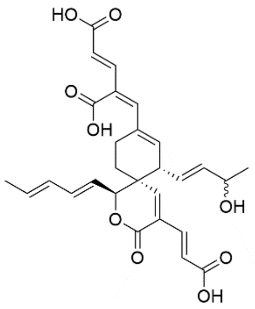

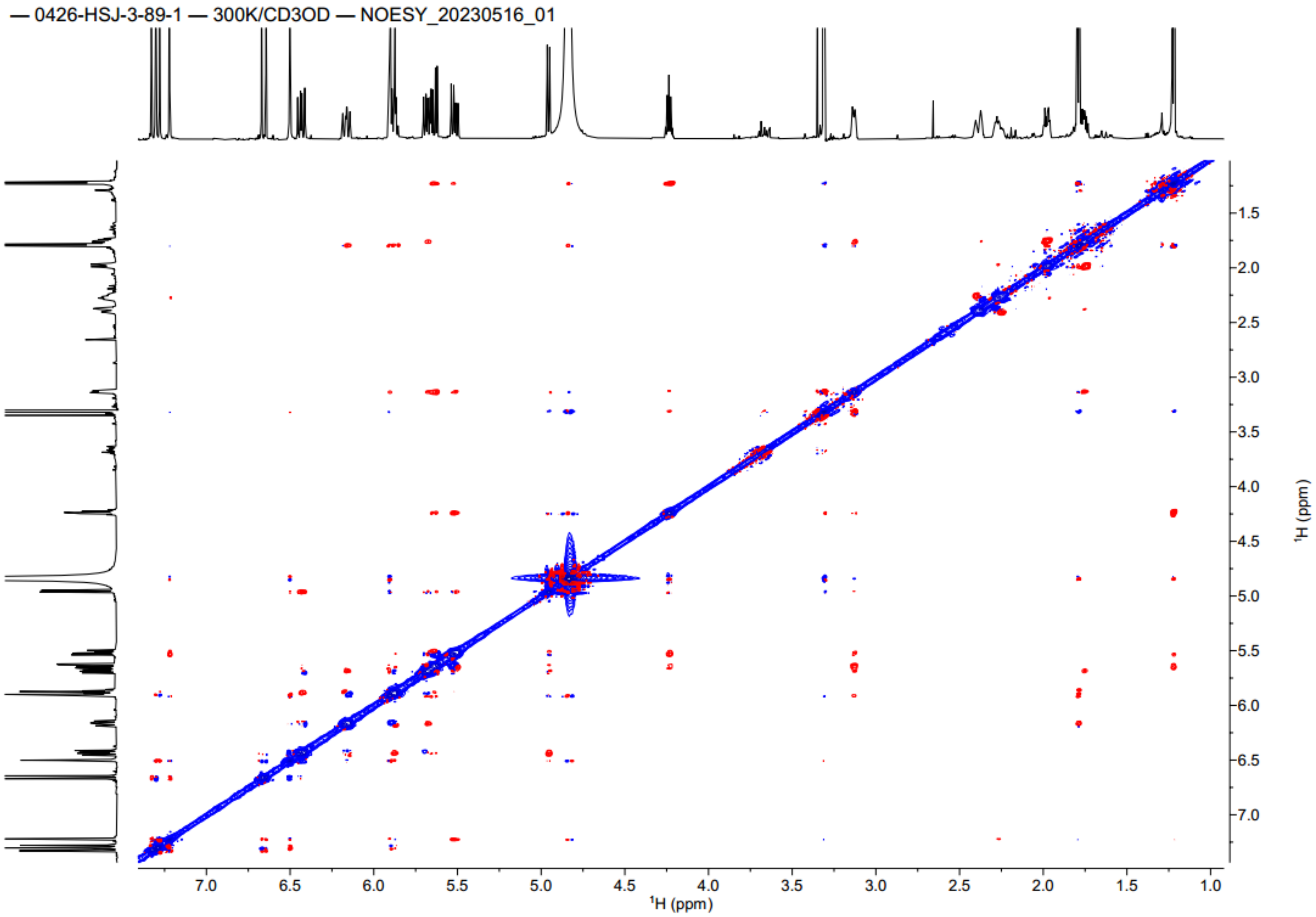

Figure S16. NOESY spectrum of compound **2**.

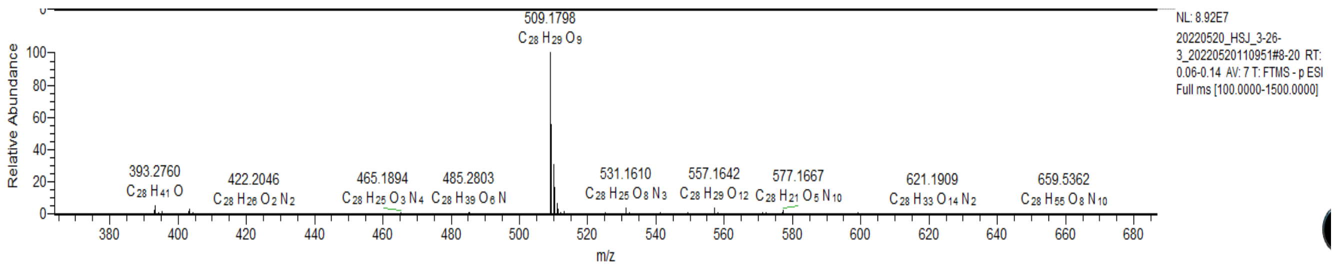

Figure S17. HRESIMS spectrum of compound **3**.

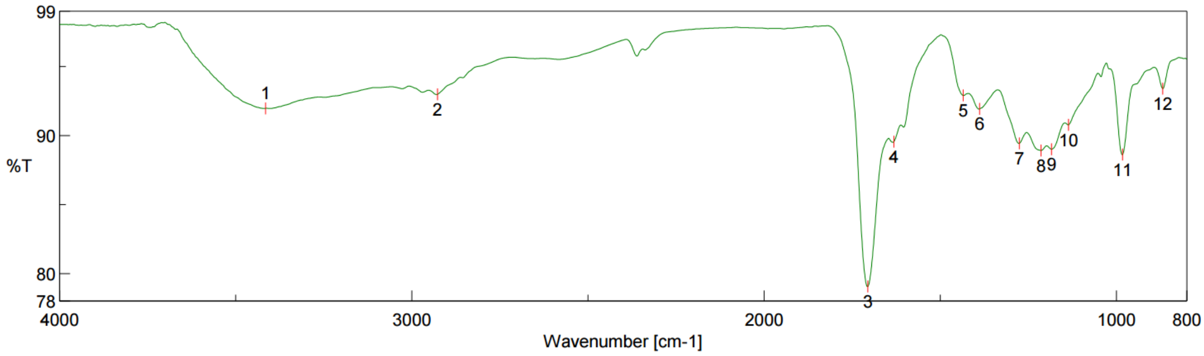

[Result of Peak Picking]

No.	Position	Intensity	No.	Position	Intensity	No.	Position	Intensity	No.	Position	Intensity	No.	Position	Intensity
1	3415.31	91.9601	2	2927.41	92.9743	3	1705.73	79.0509	4	1631.48	89.5515	5	1434.78	92.8973
6	1388.5	91.925	7	1275.68	89.4255	8	1213.97	88.9087	9	1184.08	89.0011	10	1135.87	90.7721
11	982.554	88.615	12	868.774	93.3953									

Figure S18. IR (ZnSe) spectrum of compound **3**.

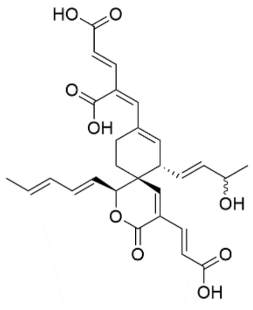

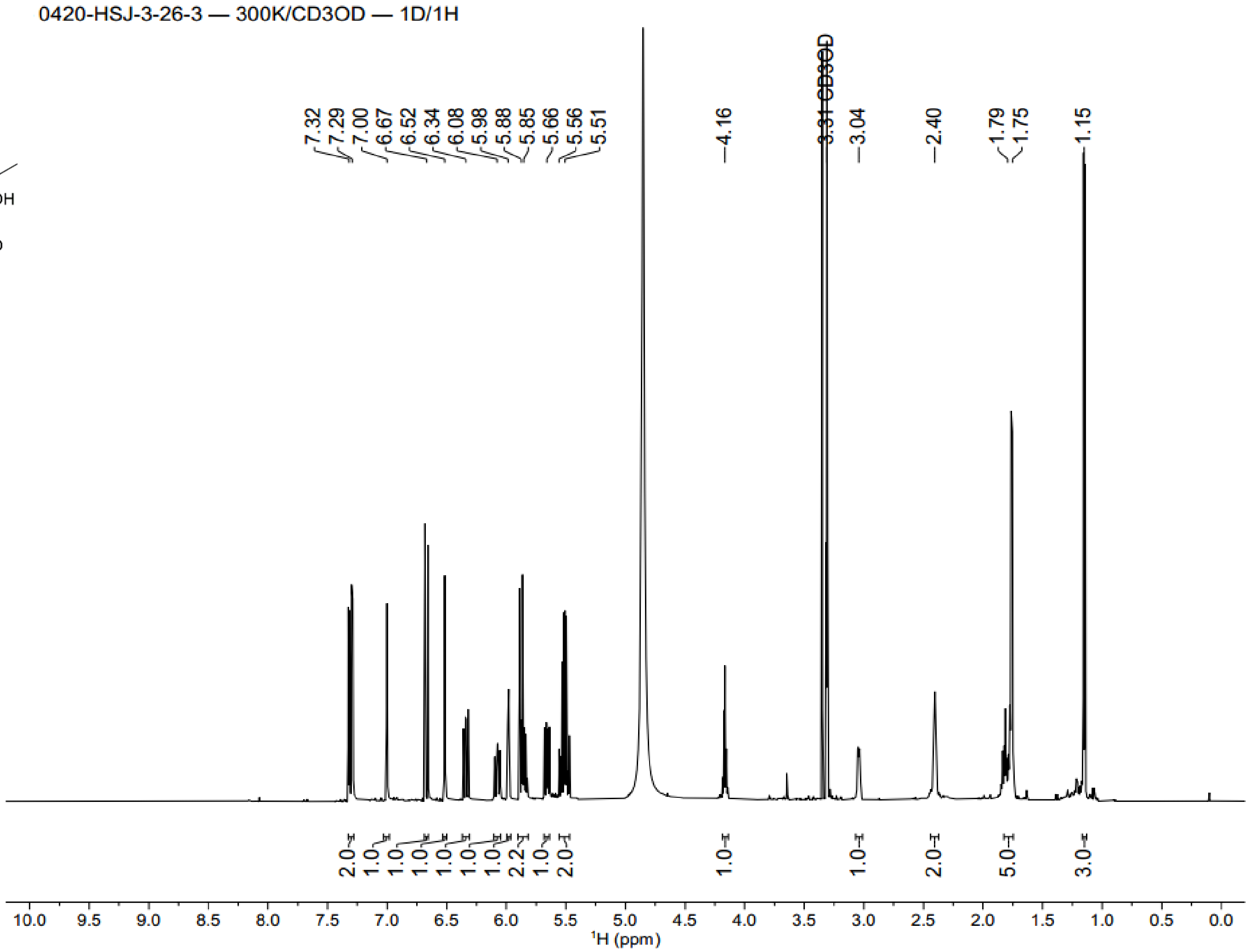

Figure S19. ^{1}H NMR (600 MHz, methanol-d_4) spectrum of compound **3**.

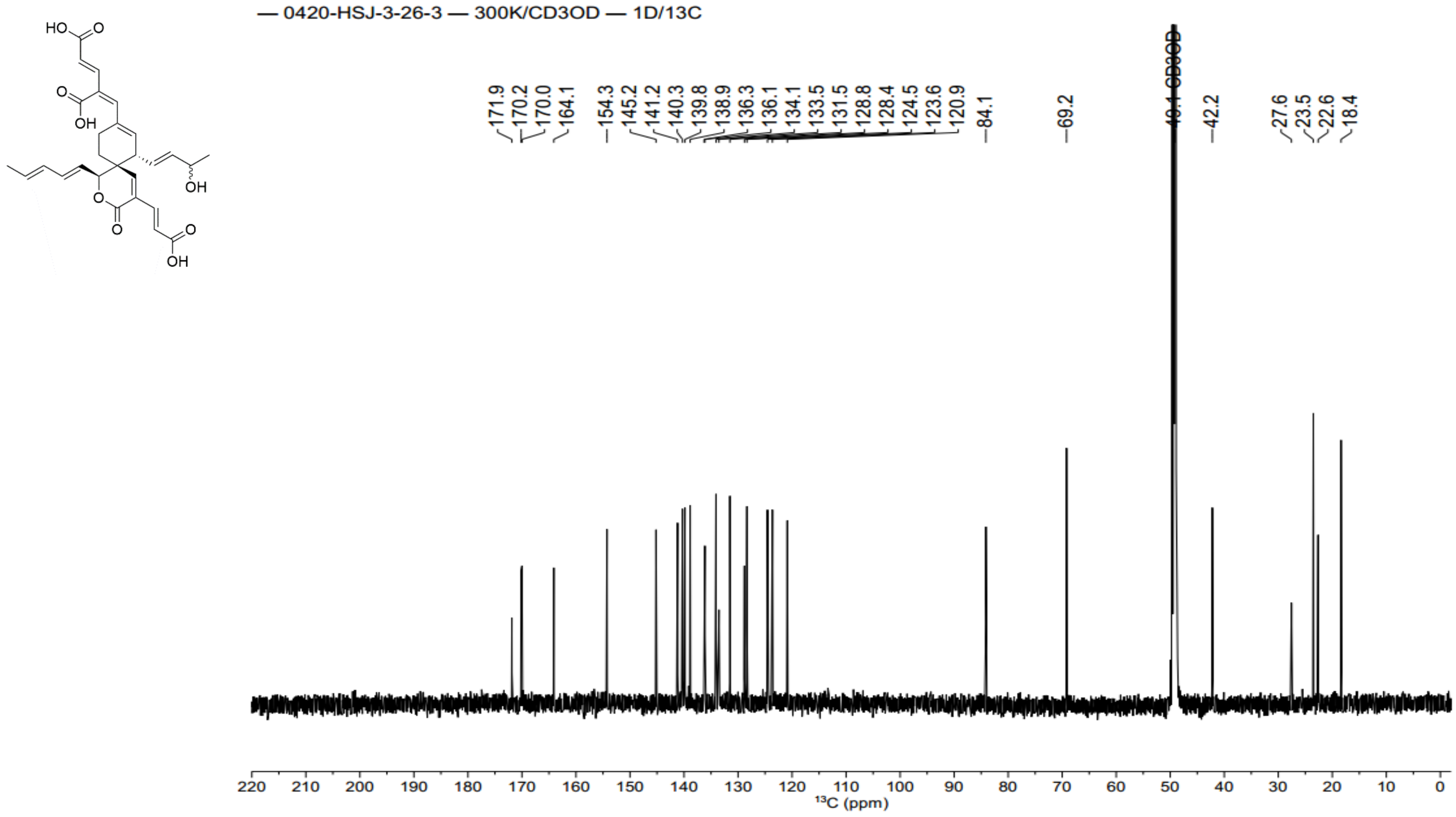

Figure S20. ^{13}C NMR (150 MHz, methanol-d_4) spectrum of compound **3**.

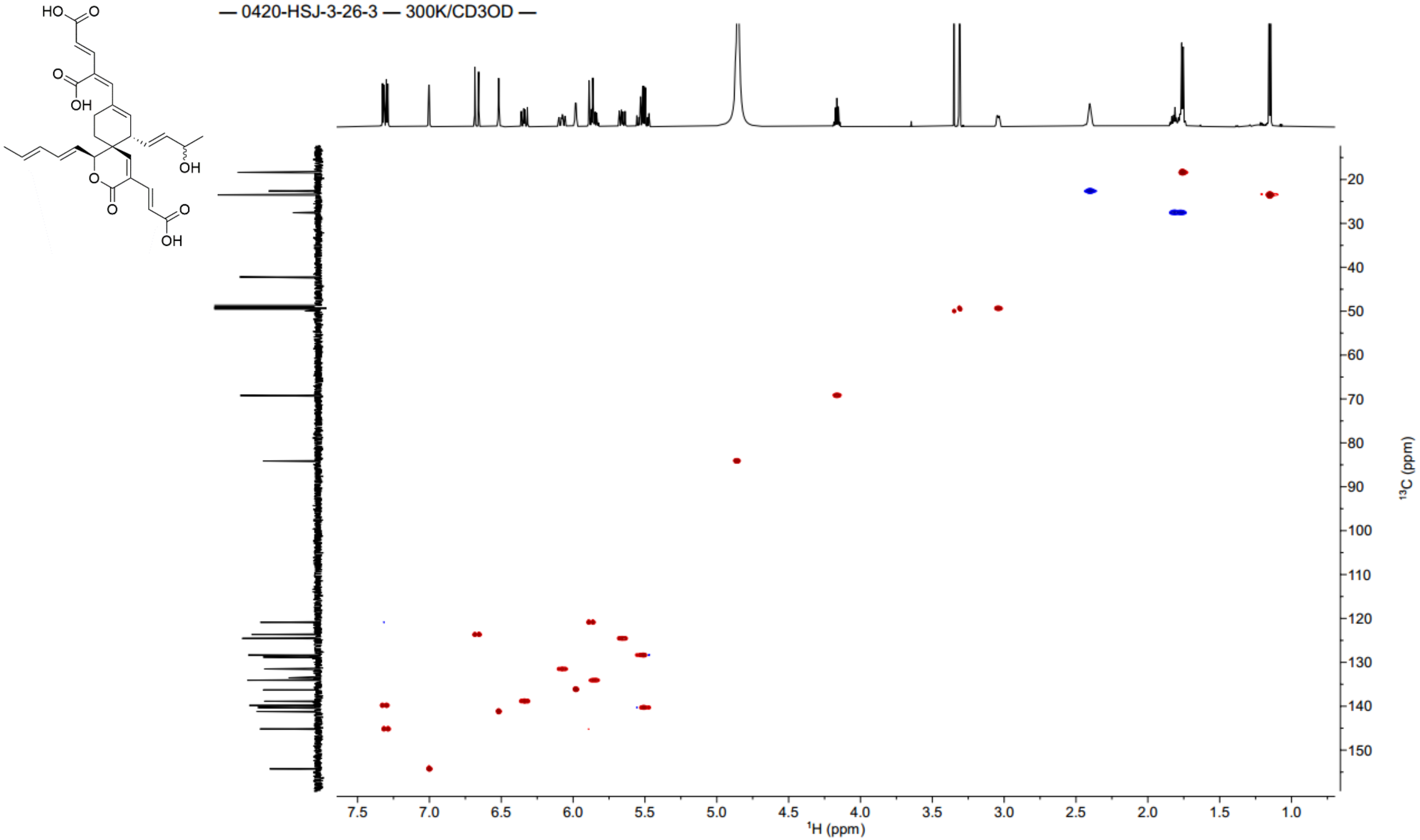

Figure S21. HSQC spectrum of compound **3**.

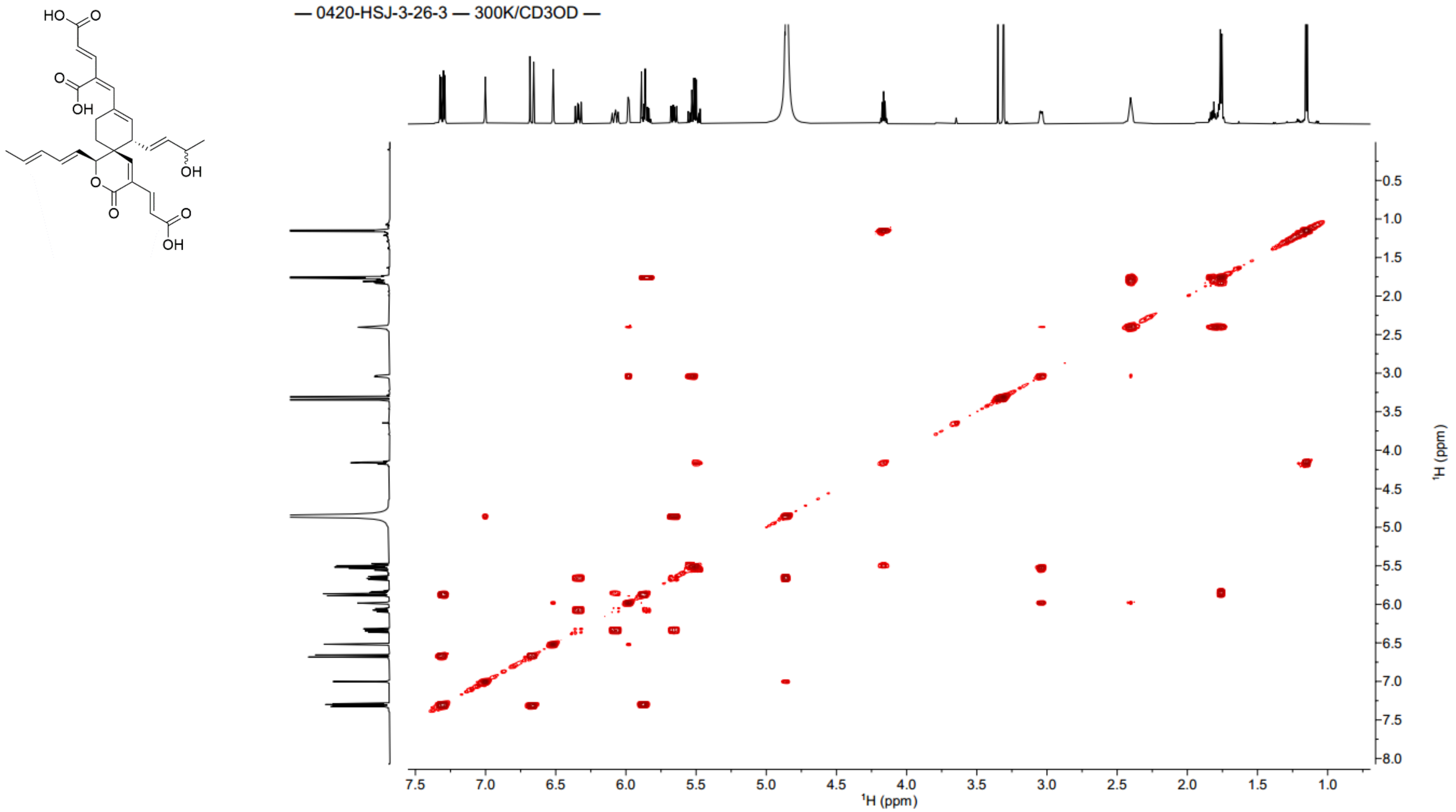

Figure S22. COSY spectrum of compound **3**.

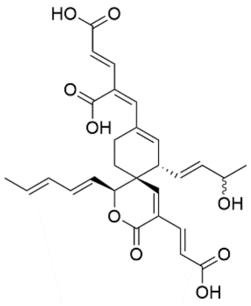

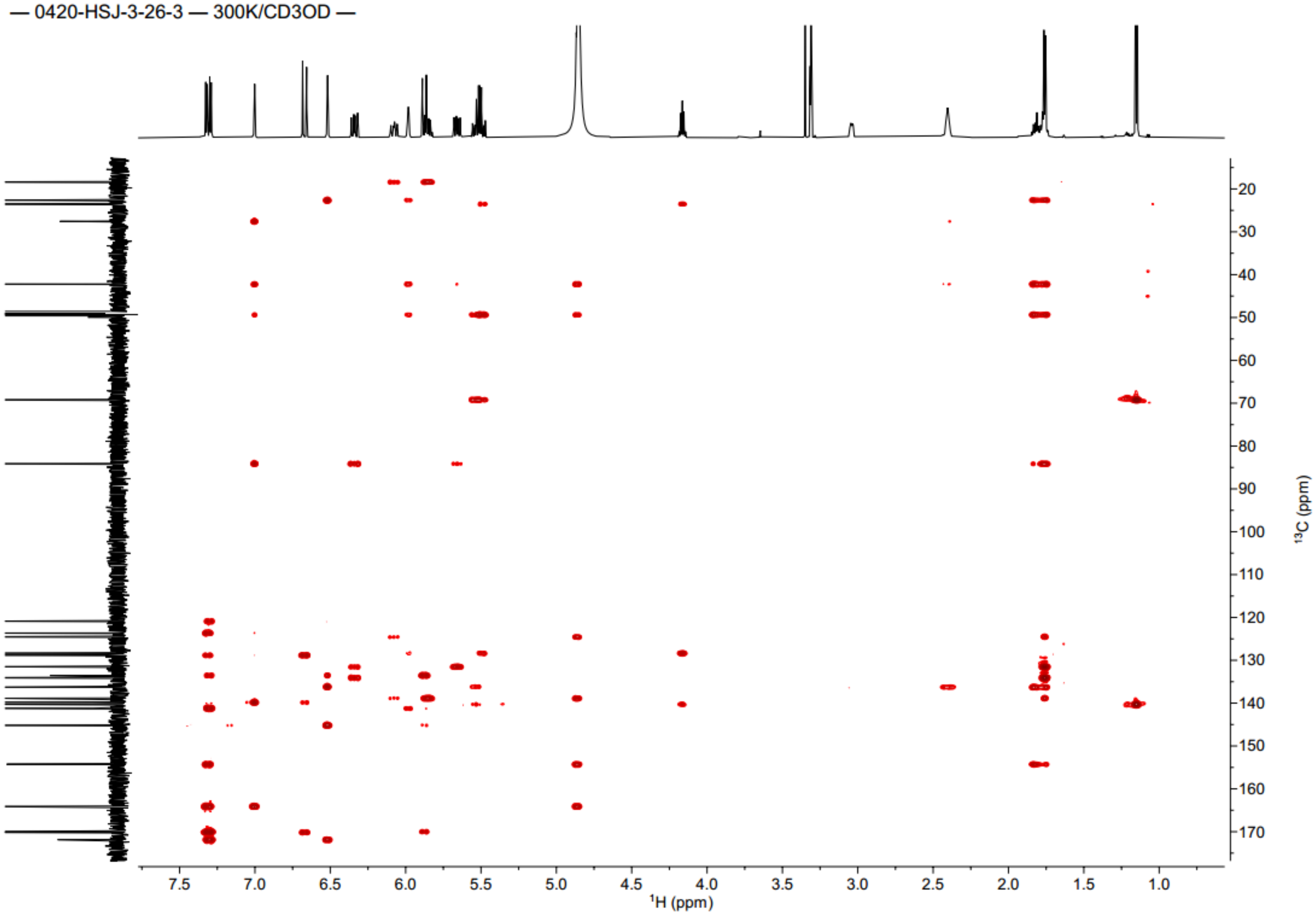

Figure S23. HMBC spectrum of compound **3**.

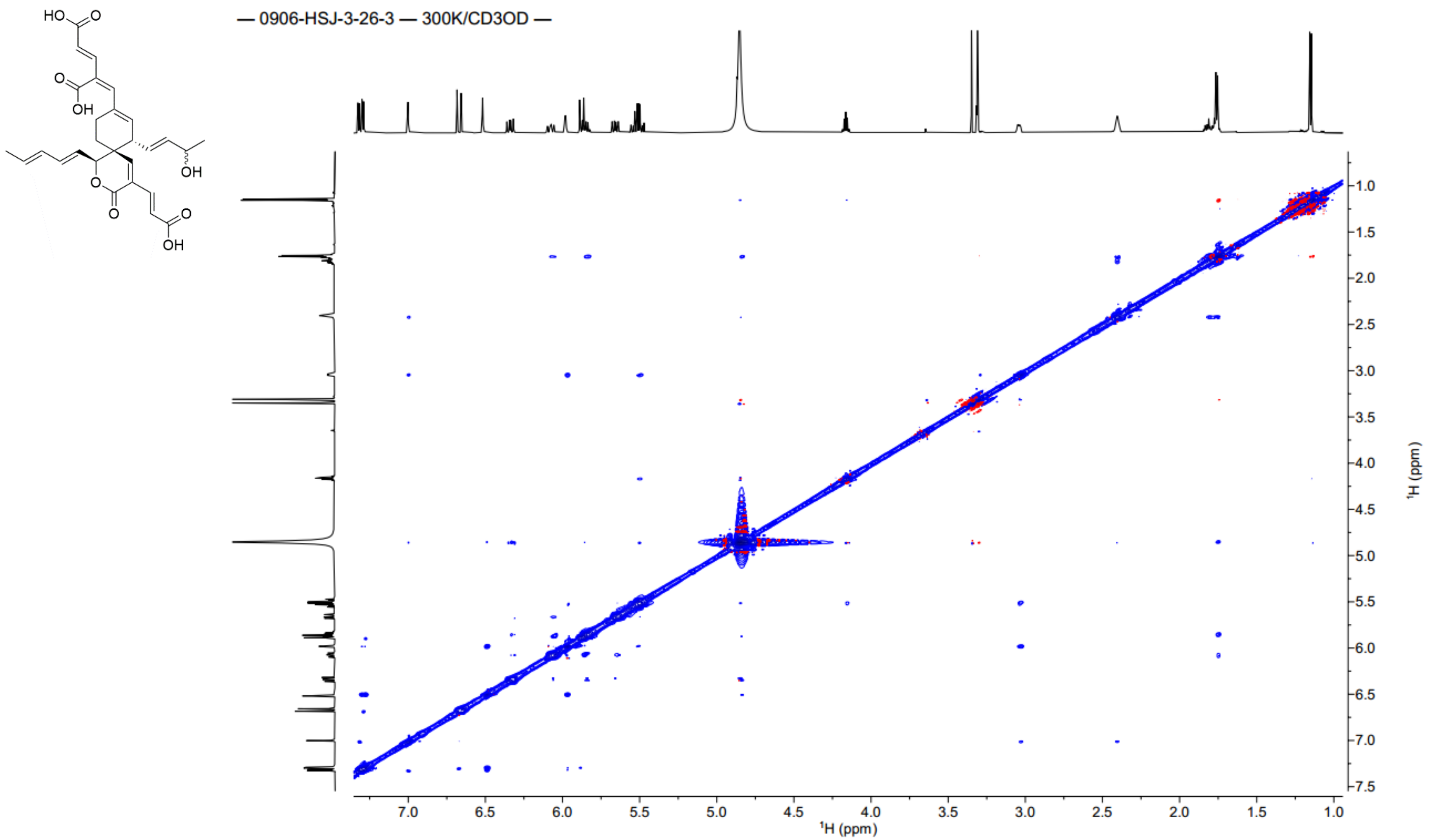

Figure S24. NOESY spectrum of compound **3**.

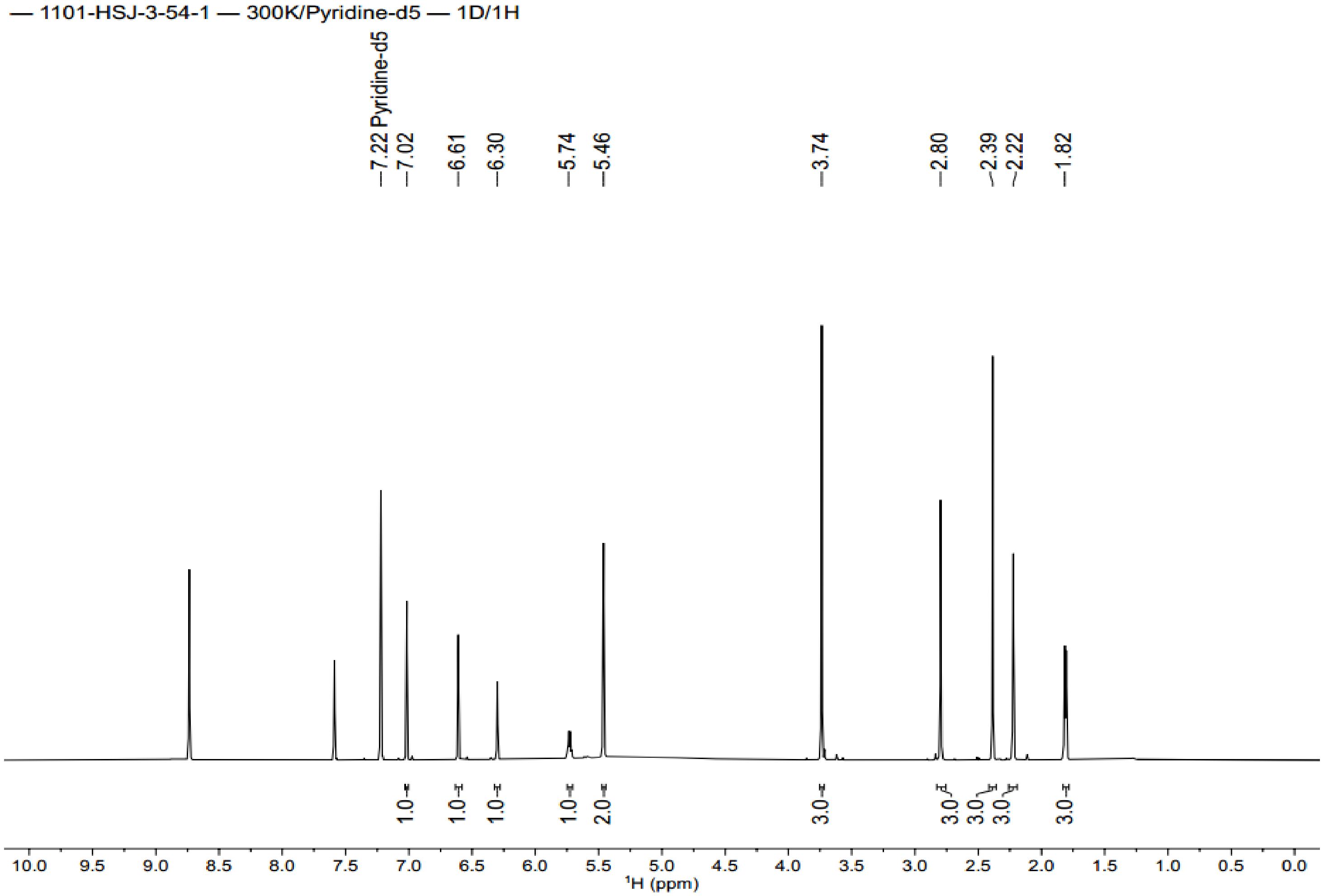

Figure S25. ^{1}H NMR (600 MHz, pyridine-d_5) spectrum of compound **4**.

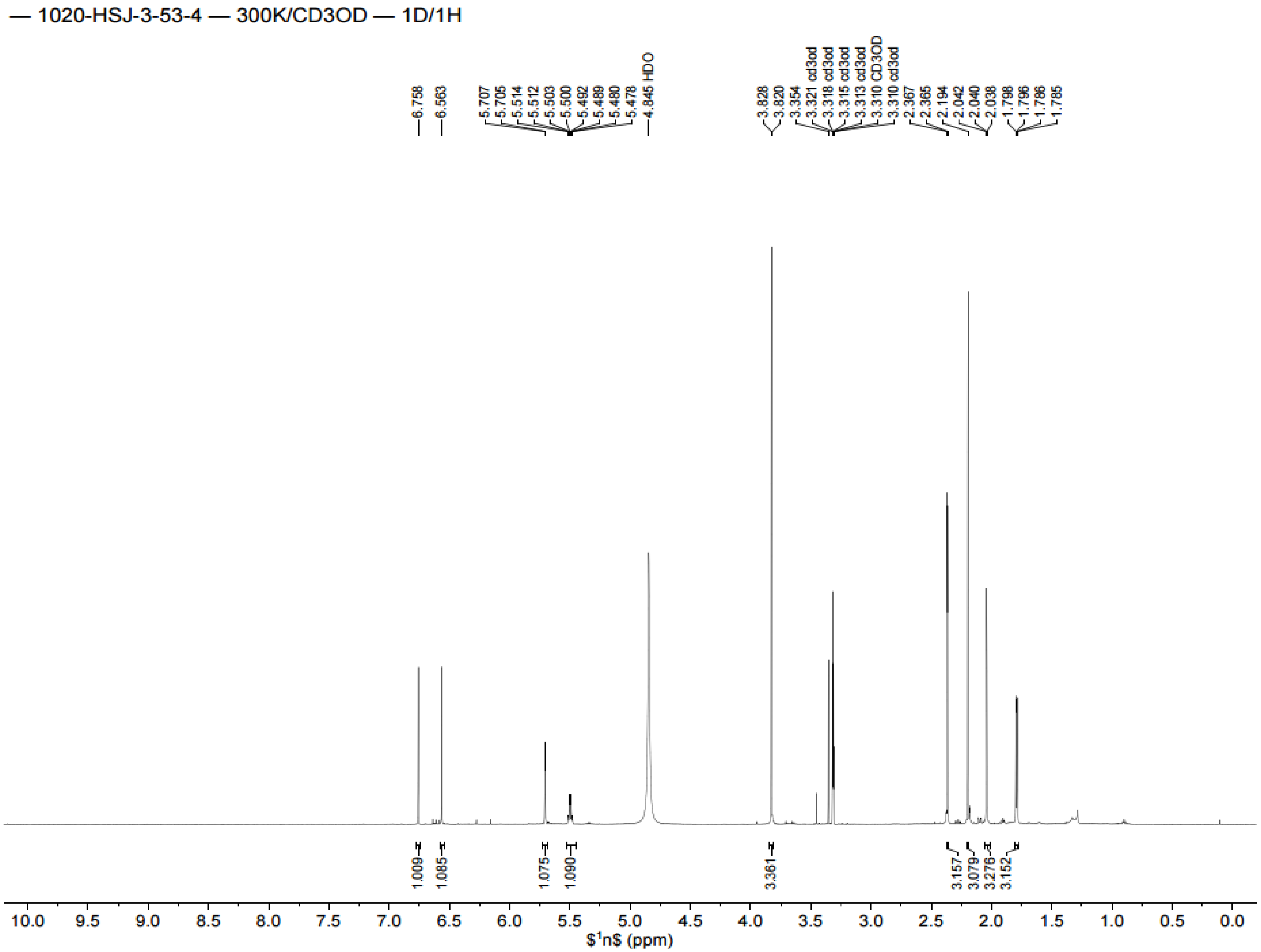

Figure S26. ^{1}H NMR (600 MHz, methanol-d_4) spectrum of compound **5**.

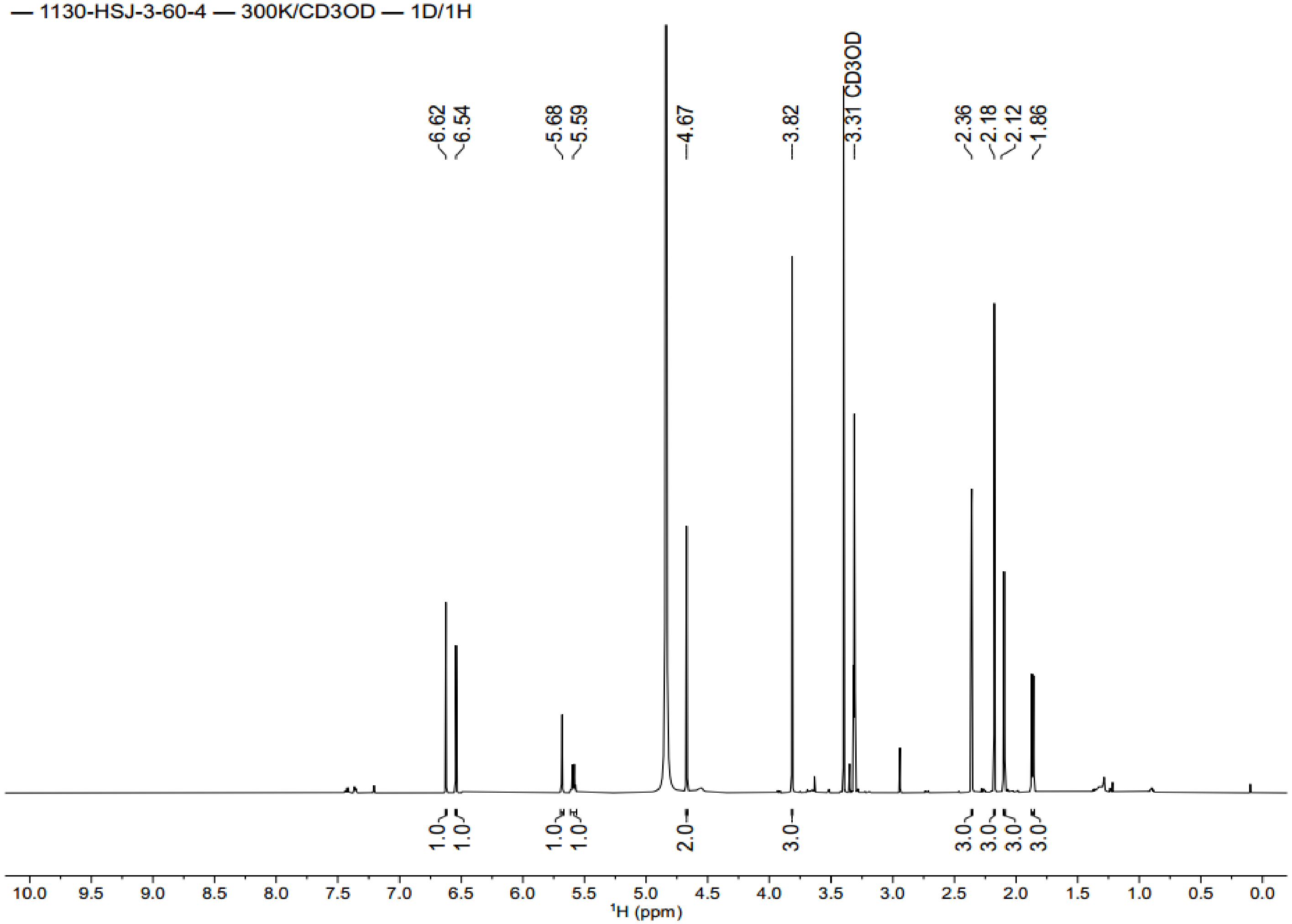

Figure S27. ^{1}H NMR (600 MHz, methanol-d_4) spectrum of compound **6**.

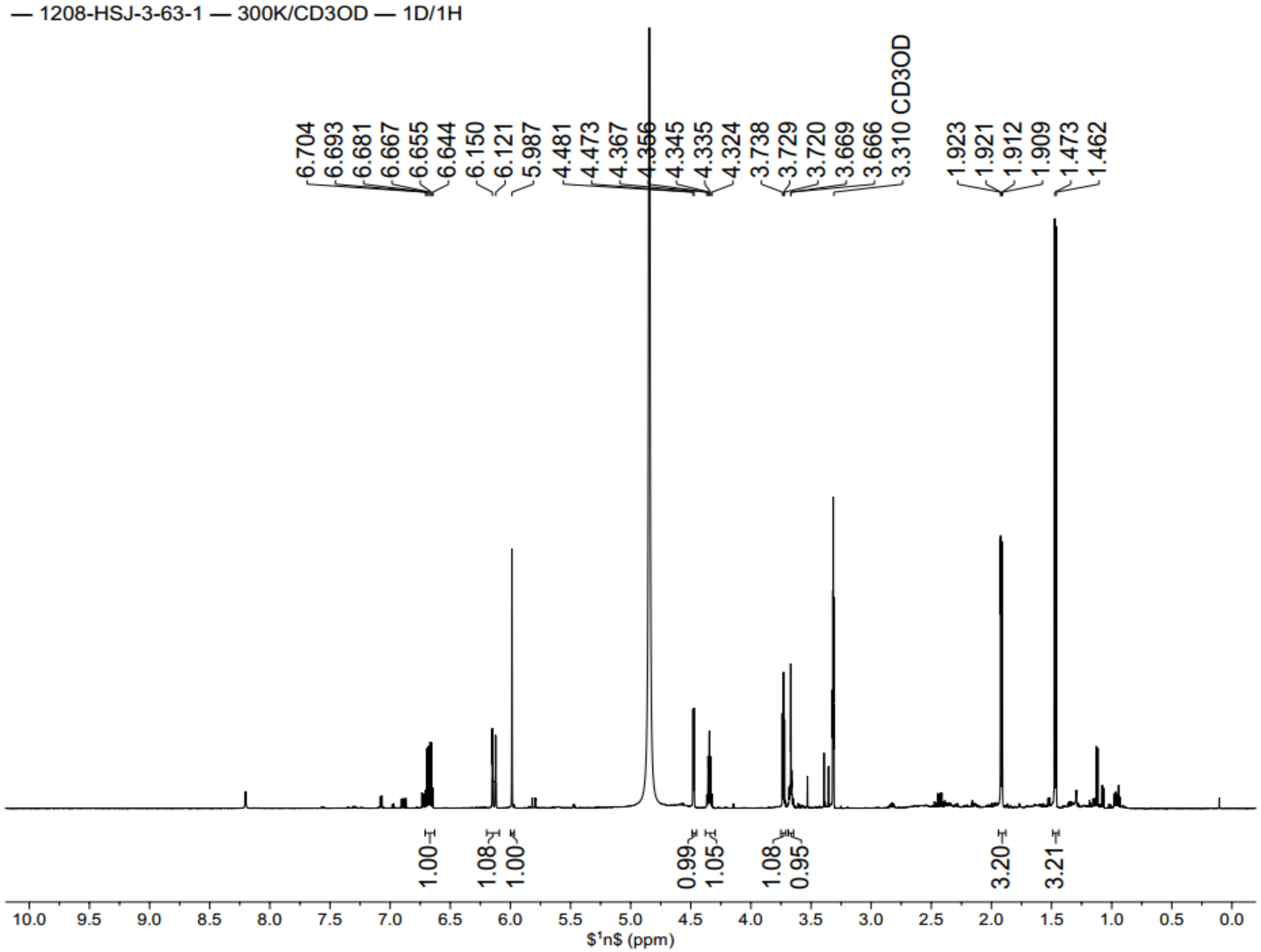

Figure S28. ^{1}H NMR (600 MHz, methanol-d_4) spectrum of compound **7**.

Table S1. CDOCKER energy and interaction pattern of compounds **1–3** and curcumin docked into iNOS (PDB ID: 1QW4).

		Compounds			
**Interactions**	**Interaction residues**	**1**	**2**	**3**	**Curcumin***
**CDOCKER interaction energy (kcal/mol)**		**-52.31**	**-76.40**	**-80.36**	**-61.23**
**Hydrogen bond**	ARG193		Yes	Yes	
	ARG260	Yes	Yes	Yes	Yes
	ASP376	Yes	Yes	Yes	
	GLN257	Yes	Yes	Yes	
	GLU371		Yes	Yes	Yes
	TYR341	Yes	Yes	Yes	Yes
	TYR367		Yes	Yes	Yes
**Salt bridge / attractive charge**	ARG260	Yes	Yes	Yes	Yes
	ARG375		Yes	Yes	Yes
	ARG382	Yes	Yes	Yes	Yes
**Pi–Pi interaction**	TRP457	Yes	Yes	Yes	Yes
**Pi–Alkyl /Alkyl**	PRO344	Yes	Yes	Yes	Yes
	VAL346	Yes	Yes	Yes	
	PHE363		Yes	Yes	Yes
	TRP340	Yes	Yes	Yes	
	TRP366			Yes	
	TRP188		Yes		
**van der Waals**	ALA191, ALA345, CYS194, MET349, ASN348, GLY196, GLY365	Yes	Yes	Yes	Yes
